# Organoid platinum-resistance model identifies KRT17 as a biomarker of targeted therapy in ovarian cancer

**DOI:** 10.1016/j.isci.2025.113999

**Published:** 2025-11-10

**Authors:** Juliane Reichenbach, Juliana Schmid, Sophia Hierlmayer, Tingyu Zhang, Ilaria Piga, Sophia Geweniger, Jonas Fischer, Aarushi Davesar, Nemanja Vasovic, Anca Chelariu-Raicu, Fabian Kraus, Alexander Burges, Bastian Czogalla, Doris Mayr, Tobias Straub, Christoph Klein, Jesper V. Olsen, Sven Mahner, Fabian Trillsch, Mirjana Kessler

**Affiliations:** 1 Department of Obstetrics and Gynecology, University Hospital, Ludwig-Maximilians-University Munich, 81377 Munich, Germany; 2 Bavarian Cancer Research Center (BZKF), 81377 Munich, Germany; 3 German Cancer Consortium, DKTK, Partner Site Munich, 69120 Heidelberg, Germany; 4 Proteomics Program, Faculty of Health and Medical Sciences, Novo Nordisk Foundation Center for Protein Research, University of Copenhagen, 2200 Copenhagen, Denmark; 5 Department of Pathology, LMU Munich, 80337 Munich, Germany; 6 Bioinformatics Unit, Biomedical Center, LMU Munich, 82152 Martinsried, Germany; 7 Department of Pediatrics, Dr. Von Hauner Children’s Hospital, University Hospital, Ludwig-Maximilians-University Munich, 80337 Munich, Germany

**Keywords:** Molecular biology, Cell biology, Cancer

## Abstract

Variable responses to platinum chemotherapy and the emergence of resistant disease drive high mortality in high-grade serous ovarian cancer (HGSOC). To study resistance mechanisms, we developed the organoid drug resistance assay (ODR-test) with patient-derived organoids from our ovarian cancer biobank and identified sustained phenotypic reprogramming and cellular plasticity of organoids under carboplatin pressure as a conserved mechanism irrespective of the basal resistance level. Transcriptional and proteomic analyses revealed changes in cell adhesion and differentiation as adaptive responses that lead to an increase in resistance. We identified Keratin 17 (KRT17) as a mediator of platinum resistance and validated its function by CRISPR-Cas9 and overexpression. Additionally, we found that KRT17 expression status (K-score) is a significant negative prognostic histopathological biomarker in a large cohort (*N* = 384) of patients with advanced HGSOC. In organoids, increased KRT17 levels enhanced sensitivity to PI3K/Akt inhibitors alpelisib and afuresertib, highlighting the potential of KRT17 as a stratification biomarker for targeted therapies.

**Video abstract:**

## Highlights

• Carboplatin triggers long-term cellular reprogramming in ovarian cancer organois
• ODR-test mimics the gradual platinum resistance increase observed in patients *in vitro*
• Rise in KRT17 drives platinum resistance and sensitizes organoids to PI3K inhibition
• Positive KRT17 status (K-score) is a negative prognostic factor in advanced ovarian cancer

## Graphical abstract

**Figure:**
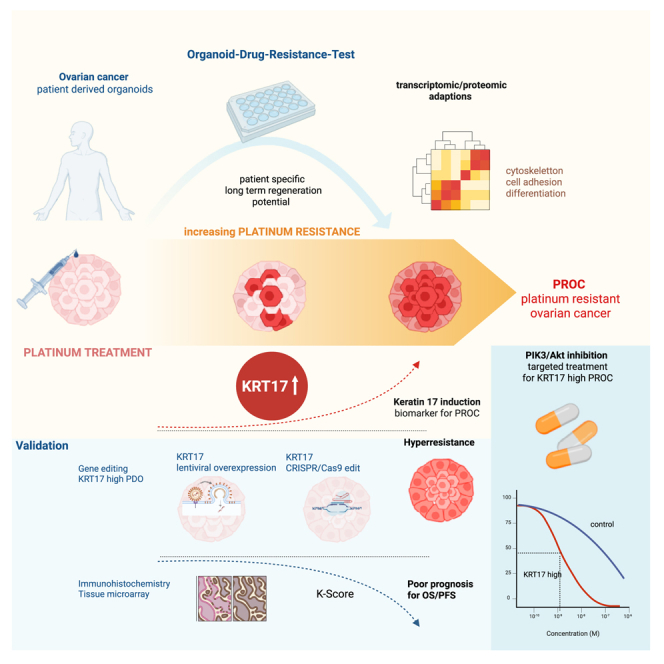

## Introduction

The pronounced heterogeneity of high-grade serous ovarian cancer (HGSOC) challenges the development of targeted therapies, leaving platinum-based chemotherapy as the main backbone of treatment.^1^ However, while initially effective, in the vast majority of patients, its therapeutic benefits diminish over time, as tumors acquire resistance, resulting in highly variable response rates among patients.^2^^,^^3^ In recurrent disease, the decision regarding platinum re-administration still relies only on clinical and chronological criteria due to the absence of molecular predictive and stratification tools.^4^ Consequently, many patients receive ineffective therapy, and therapeutic options remain limited.^5^ Despite extensive research into the molecular adaptations that tumors undergo during platinum treatment, preclinical studies have not yet yielded clinically applicable results.^6^^,^^7^ As an important advancement to standard cell lines and cell line-based xenograft models, patient-derived organoids (PDOs) of HGSOC maintain the epithelial structure, allow long-term cultivation, and preserve the full genomic and functional hallmarks of ovarian cancer.^8^^,^^9^^,^^10^^,^^11^ Thus, an HGSOC PDO biobank from a large heterogeneous prospective patient cohort with well-documented clinical data provides a unique resource for studying individual differences in platinum response and the emergence of resistant phenotypes.^12^

Standard viability-based *in vitro* drug assays quantify only the direct cytotoxicity of chemotherapy by measuring the cell viability after exposure.^13^ However, since therapy in patients likely drives selection mechanisms within a tumor that allow the expansion of pre-existing minor clones, viability testing is inadequate for capturing this critical aspect of drug response.^14^^,^^15^

Understanding the role of changes in epithelial differentiation and cell biology of tumors over time is critical to unraveling the mechanisms of platinum resistance. Transcriptional profiling of the fallopian tube epithelium, the origin of HGSOC,^16^ revealed subclusters of cells with stem cell signatures.^17^^,^^18^^,^^19^^,^^20^ Among these, the type I intermediary cytoskeletal filament protein Keratin 17 (KRT17) has been identified as one of the markers of the progenitor population.^17^^,^^18^^,^^19^^,^^20^ In other epithelial tissues, KRT17 is studied for its role in tissue regeneration and stress responses.^21^^,^^22^ By activating the PI3K/Akt signaling cascade, KRT17 contributes to keratinocyte growth and altered cell fate decisions in the context of tissue repair.^23^ Upregulated in various epithelial cancers, including HGSOC, KRT17 correlates with poor clinical outcomes, cell proliferation, and therapy resistance.^24^^,^^25^^,^^26^^,^^27^^,^^28^^,^^29^^,^^30^^,^^31^^,^^32^^,^^33^^,^^34^ Notably, the inhibition of the PI3K/Akt signaling cascade has been proposed to target chemoresistance in ovarian cancer.^35^^,^^36^^,^^37^

In this study, we have developed the *in vitro* organoid drug resistance test (ODR-test), to quantify the effect of chemotherapy, in this case, platinum, on the long-term renewal potential of organoids. Long-term cultivation of post-platinum-treated HGSOC PDOs enabled transcriptional and proteomic analyses of adaptive changes under therapeutic pressure. We suggest KRT17 as a mediator of platinum response and validated our findings of a potential clinical prognostic impact in a large cohort of patients with advanced HGSOC. Notably, organoids that overexpressed KRT17 showed improved sensitivity to the PI3K inhibitor alpelisib and the Akt inhibitor afuresertib. These observations raise the possibility that KRT17 could serve as a marker of PI3K/Akt inhibitor responsiveness. This could, in the future and after additional validation in clinical cohorts, contribute to biomarker-based treatment strategies that improve the management following suboptimal responses to platinum chemotherapy.

## Results

### The organoid-drug-resistance test

**Figure 1:**
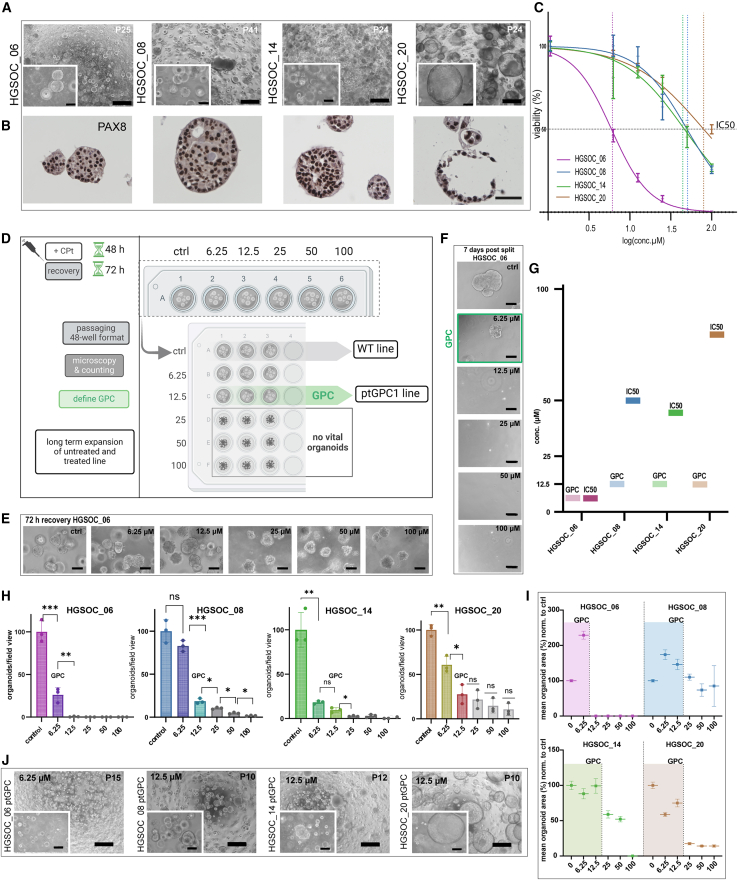
Organoid drug resistance (ODR) test (A) Phase contrast images of four PDO lines. Scale bars, 500 μm for the overview image and 100 μm for the zoomed view (lower left). (B) Immunohistochemistry images show strong PAX8 expression and characteristic tumor morphology, loss of polarity, and polymorphic nuclei. Scalebar, 50 μm. (C) Combined log-transformed dose-response curves showing patient-specific differences in sensitivity to carboplatin. Error bars represent mean ± SEM of technical triplicates. Platinum IC50 concentration (μM). The dotted line indicates 50% luminescence for the respective line. Drug response curves are representative of at least two independent drug response tests in each donor line. (D) Schematic ODR-test workflow illustrates key experimental steps of the assay. (E) Phase contrast images of representative PDO line HGSOC_06 at 72 h recovery and (F) 7 days post-split (P1) for each concentration (scale bars, 100 μm), green box = GPC for the given line, where long-term expansion was possible. (G) Direct comparison of GPC and IC50 levels in μM for each PDO line. (H) Diagram of normalized organoid counts in P1. Error bars represent mean ± SEM of technical replicates from different wells. (I) Mean organoid surface area diagrams show the distribution of organoids that regrew after the carboplatin challenge. The range is calculated as mean ± SEM. (J) Phase contrast images of 4 ptGPC PDO lines, illustrating sustained long-term expansion >3 months, after ODR test. Scale bars, 500 μm and 100 μm for the zoomed view (lower left). P-values are calculated by the Student’s t test (∗*p* < 0.05, ∗∗*p* < 0.01; ∗∗∗*p* < 0.001, ∗∗∗∗*p* < 0.001).

Primary tumor tissue from patients with HGSOC, obtained during ovarian cancer debulking surgeries, was processed, and patient-derived organoid (PDO) lines were generated within our ovarian cancer organoid biobank.^12^ To investigate patient-specific platinum responses, we selected four organoid lines (HGSOC_06, HGSOC_08, HGSOC_14, and HGSOC_20) from chemotherapy-naive patients with documented clinical backgrounds (Table S1). All PDO lines demonstrated stable growth patterns, maintaining stable long term growth potential *in vitro*, with expandability over 20 passages for more than six months (Figure 1A). Analysis of cellular architecture and expression of HGSOC hallmark genes by immunhistochemistry confirmed strong PAX8 positivity, presence of a TP53 mutation, and changes in epithelial organization characteristic of transformed phenotypes (Figures 1B and S1A).

Initially, we quantified the response of organoids to carboplatin by measuring the change in cell viability after 72 h, normalized to the control, and determined IC50 values for each patient-specific organoid line (Figure 1C). The IC50 represents the concentration of carboplatin required to achieve a 50% reduction of cell viability.

Notably, PDOs consist of cells at various stages of differentiation, with the progenitor population playing a key role in long-term cancer growth. Given that the residual growth potential of chemotherapy-surviving cancer cells presents the main challenge for strategies preventing recurrence, we focused on identifying sustained changes in organoids caused by sublethal carboplatin exposure. Therefore, we have designed the organoid drug resistance (ODR) test to identify and quantify the potential changes in renewal capacity as a consequence of drug treatment. In brief, PDO lines were exposed for 48 h to carboplatin, at five different concentrations (6.25 μM, 12.5 μM, 25 μM, 50 μM, and 100 μM) alongside a control, followed by a 72 h recovery phase in fresh medium and were subsequently passaged (P1) to a multiwell format in technical triplicates (Figure 1D). We selected a 48 h exposure because this duration more closely reflects the limited plasma half-life of carboplatin in patients. While signs of acute cellular stress were notable at the 72 h recovery (P0) (increase in granularity, dark appearance, loss of adhesion, Figures 1E and S1B), the functional impact of carboplatin on the organoid regeneration potential could be assessed only after singularization and reseeding (Figure 1F). The growth permitting concentration (GPC) was defined as the highest concentration of carboplatin at which organoids maintained their progenitor potential after splitting, assessed by their non-impeded growth and serving as a measure of tumor intrinsic platinum resistance. Compared to the previously measured IC50 values, the GPCs determined by the ODR-test were in three cases substantially lower (Figure 1G), despite shorter treatment time. This implies that for these tumors, a much lower platinum concentration is sufficient to exhaust long-term growth potential than the one necessary to reduce overall viability to 50%. We observed a consistent trend of decreasing organoid formation potential with increasing concentrations of carboplatin in all donors. The number and size of the organoids in P1 were quantified by analysis of phase contrast images from independent wells in technical replicates. The magnitude of the reduction in organoid count in P1 varied markedly between the lines, indicating substantial individual differences in their capacity to withstand platinum challenge (Figure 1H).

The surface area of individual organoids was determined with the Qupath software annotation tool^38^ (Figures S1C and S1D). Surface size distribution analysis revealed that, despite a reduction in organoid formation efficiency, the mean size of the organoids that regrew in P1 remained stable until the GPC was reached (Figure 1I). In line with this, at concentrations higher than the GPC, organoids were smaller and could not be further expanded (Figures 1I and S1E). Although initially viable, these 3D cellular aggregates effectively lost regeneration potential and exhibited growth arrest two to three weeks after carboplatin exposure. Importantly, organoids that regrew in P1 at the GPC concentration despite reduced count could be maintained in long-term culture (>3 months) and exhibited expansion potential comparable to that of non*-*treated controls (Figure 1J). In conclusion, ODR testing identifies a critical concentration of platinum (GPC) required to effectively exhaust the progenitor potential of HGSOC organoids. Average organoid size, rather than organoid count, after the challenge is a more suitable criterion to predict long-term growth capacity. Importantly, the generation of ptGPC1 lines (post-treatment at GPC growing) was successful in all cases, with continued stable growth in culture, confirming the full preservation of growth capacity after carboplatin treatment.

### Phenotypic reprogramming in post-platinum organoids

**Figure 2:**
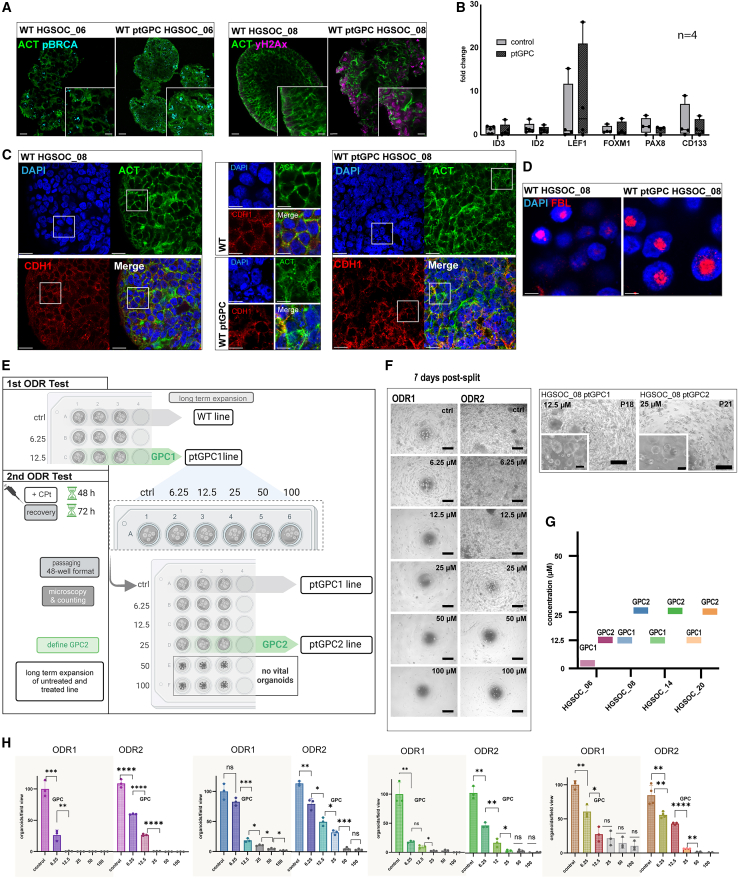
*In vitro* modeling of platinum resistance (A) IF staining of *p*-BRCA (left, cyan) and γ-H2AX (right, magenta) of untreated and ptGPC PDOs showing sustained DNA damage and activation of DNA repair in pre-exposed lines, representative of findings from two different wt/ptGPC donors (HGSOC_06 and HGSOC_08). ACT = Actin by phalloidin, green. Scale bars, 20 μm and 10 μm, lower right. (B) qPCR of expression of stemness- and differentiation-related target genes in ptGPC organoids (*n* = 4 independent donor lines, wt and ptGPC pairs). Data are normalized to the average expression of the respective genes in the control group. Error bars, mean ± SEM. (C and D) IF staining shows changes in the organization of cytoskeleton (Actin by phalloidin, green and E-Cadherin (CDH1, red in C), nuclear morphology (DAPI, blue), and nucleolus (fibrillarin, red in D) in untreated vs. ptGPC PDOs of line HGSOC_08. Scalebars, 20 μm (left and right), 10 μm (middle), for C, and 5 μm for D. Data are representative of images from three different staining experiments. (E) Schematic ODR2-test workflow for the rechallenge of the ptGPC1 organoids. (F) Direct comparison of day 7, P1 phase contrast images, and long-term expansion, for the representative donor line after ODR1 and ODR2 test, showing differences in sensitivity/growth potential (scale bars, 500 μm, and 100 μm) leading to (G), increasing GPC (GPC1 to GPC2 comparison in μM), for four pairs of PDO lines. (H) Quantification of the change in drug sensitivity, measured by organoid count, normalized to the control for ODR1 and ODR2. Scale bars are ±SEM of technical triplicates, P-values are calculated by the Student’s t test (∗*p* < 0.05, ∗∗*p* < 0.01; ∗∗∗*p* < 0.001, ∗∗∗∗*p* < 0.0001).

The robust expandability of the ptGPC1 lines after the ODR-test provided the opportunity to compare them to the respective untreated control PDO lines. The repair process of the double-stranded DNA breaks is coordinated by phosphorylated Histone protein A2X (γH2AX), initiating the recruitment of other repair proteins to the damage site, including BRCA1.^39^^,^^40^ Notably, although more than two months passed since the initial carboplatin treatment, ptGPC1 PDO lines still exhibited sustained elevated γH2AX and pBRCA1, as detected by confocal imaging (Figure 2A). Meanwhile, expression levels of pathways associated with maintenance of progenitor potential, including Wnt- and BMP-target genes, as well as regulators of lineage fidelity and differentiation (*PAX8*, *FOXM1*, and *CD133*), analyzed by qPCR, showed no consistent changes in regulation among the different WT/ptGPC1 line pairs (Figure 2B). The expression levels of *ID2* and *ID3*, which are downstream of active BMP signaling, remained relatively stable and robust, as indicated by low ΔCt values (Figure S1F). This aligns with the preserved growth capacity of ovarian cancer PDOs, as BMP activity is known to promote their proliferation.^8^^,^^12^ Despite preserved growth capacity, ptGPC1 organoids showed changes in cellular architecture visualized by actin stain phalloidin as well as prominent defects in the morphology of the nuclei (Figure 2C). The expression pattern of the nucleolus marker fibrillarin was found to be a more condensed and larger area in comparison to controls (Figure 2D). These findings suggest that carboplatin induces irreversible molecular changes in organoids without impairing the general ability of cell proliferation and progenitor potential.

To further evaluate whether the observed cellular phenotype in ptGPC1 organoids plays a functional role in response to the platinum challenge, we subjected them to a second round of carboplatin treatment and ODR testing (ODR2-test) using the same workflow (Figure 2E). We found that organoids that underwent re-challenge showed notably improved formation efficiency and growth potential after the second treatment when compared with the outcomes from ODR1-test (Figures 2F and S2A–S2D). For all tested lines, the growth-permitting concentration of the second challenge, defined as GPC2, surpassed the previous GPC1 value (Figure 2G). Importantly, for each line and at every carboplatin concentration, organoid counts were consistently higher post-ODR2 compared to post-ODR1 counts, resulting in enhanced ODR-response profiles (Figure 2H). In summary, repeated *in vitro* platinum treatment and ODR testing revealed the gradual development of resistance in PDO organoids, including those derived from patients with clinically favorable platinum responses.

### Cytoskeleton remodeling promotes platinum resistance

**Figure 3:**
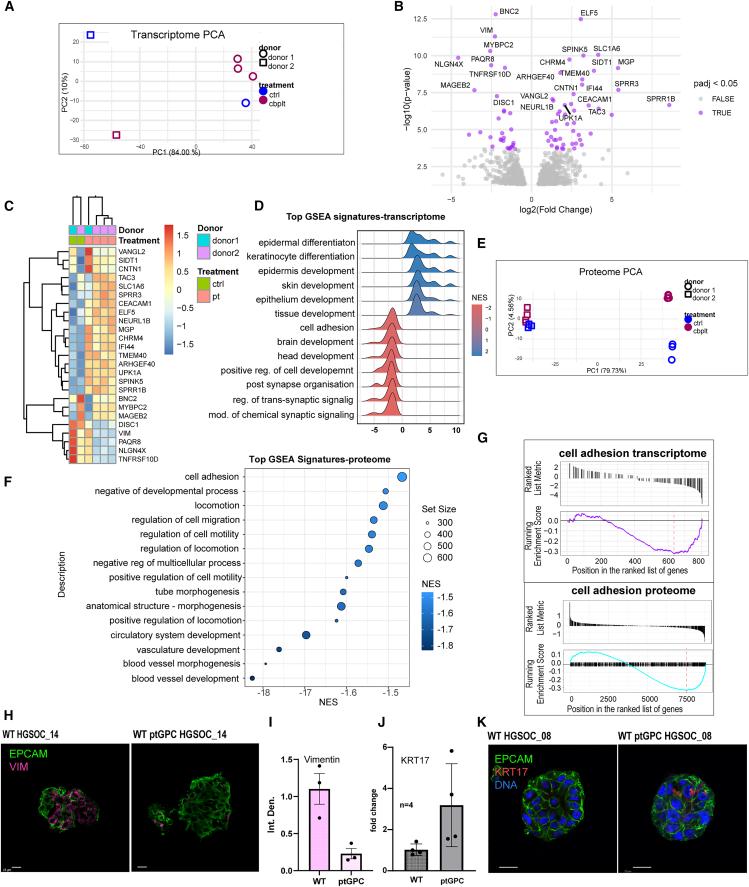
Post-platinum organoid transcriptomic and proteomic characterization (A) PCA plot of mRNA sequencing of control vs. GPC lines, for two donors, showing patient-specific characteristics dominating expression profiles of the organoids. (B) Volcano plot of top 25 regulated candidate genes (Log2 Foldchange, p-adjust <0.05). (C) Heatmap analysis of the top 25 regulated genes (p-adjust <0.05) illustrates that, despite great differences between donors, samples cluster according to the treatment. (D) GSEA for GO Biological processes in untreated vs. ptGPC1 lines identifies enrichment in pathways regulating the cytoskeleton and differentiation. (P-adjust <0,05). (E) PCA plot of mass spectrometry measurements (*n* = 4 independent technical replicates for two sets of lines, ctrl vs. GPC, total 16 samples), confirms that patient-specific characteristics are defining components in the global proteome profile. (F) GSEA of top enriched pathways for proteome (NES) identifies enrichment in downregulation for pathways regulating cell adhesion and epithelium development. (G) ES plots for cell adhesion in transcriptome and proteome data. (H) Loss of vimentin (magenta) IF staining visualized by confocal imaging in ptGPC HGSOC_14 organoids in comparison to the control line. Scale bar, 25 μm. (I) Quantification plot, showing differences in mean fluorescence intensity of different fields of view. Error bars, mean ± SEM. (J) qPCR validation of the induction of *KRT17* (red) in ptGPC1 organoids (*n* = 4 independent biological samples, Error bars, mean ± SEM) and (K), the confocal image representative of stainings from two different WT/ptGPC pairs, confirms an increase in KRT17 expression. Error bars, mean ± SEM. Scalebar, 20 μm.

Next, we aimed to gain a deeper understanding of the biology of ptGPC organoid lines by evaluating their transcriptomic and proteomic landscapes to obtain a global view of the therapy-driven long-term changes. Bulk RNA sequencing of two paired donor PDO lines (HGSOC_06 and HGSOC_08), with additional biological replicates (non-treated and ptGPC lines), revealed a strong patient-specific component in the expression profiles, as visualized by principal component analysis (PCA), with carboplatin treatment not having a defining impact on the overall gene expression profile (Figure 3A). This great interpatient heterogeneity is in line with previously published scRNA data from ovarian cancer tumor tissue.^41^ Differential expression analysis performed with the DESeq2 R package identified 837 genes differentially regulated in ptGPC1 lines (*p*-value <0.05), among which 80 were significant in the p-adjust <0.05 analysis (Table S2). The Volcano plot of the differentially expressed genes (non-treated vs. ptGPC lines) revealed the most strongly regulated candidates (Log2Foldchange and *p*-value) in response to chemotherapy (Figure 3B). Despite a pronounced difference between the two donors, reflected also in their clinical background, the set of genes showed a consistent pattern of change in expression driven by carboplatin treatment. As illustrated by heatmap analysis of the top 25 regulated genes based on their significance (p-adjust <0.05), samples clustered according to the treatment, confirming the existence of a common signature response of the organoids upon carboplatin exposure (Figure 3C). The Gene Set Enrichment Analyses (GSEA) of regulated genes for GO (gene ontology): Biological Processes revealed significant positive enrichment or upregulation in gene sets involved in tissue and epithelial development and differentiation, while cell adhesion regulators were found downregulated in ptGPC1 organoids (Figure 3D). Mass spectrometry analysis of the protein lysates of the same pairs of organoid lines in quadruplicates confirmed key findings of the transcriptional profiling, with highly similar sample distribution in the PCA plot (Figure 3E). Likewise, GSEA for Biological Processes showed significant enrichment in pathways regulating migration and cell adhesion (Figure 3F). On the proteome level, a total of 947 (p-adjust <0.05) proteins were confirmed to be regulated in the same fashion in both donors (Table S3). The enrichment score plots (ES) for the downregulation of adhesion-related signatures in transcriptomic and proteomic datasets yielded comparable results, providing complementary evidence obtained by independent omics methodologies of the downregulation of adhesion complexes in ptGPC organoids (Figures 3F and 3G).

Remodeling of the cytoskeleton and changes in adhesion have previously been found to promote therapy resistance. The downregulation of vimentin, one of the main regulators of mechanosensing,^42^ has been reported in ovarian cancer cell line models during the acquisition of resistance.^43^^,^^44^ Accordingly, a reduction of vimentin signal intensity was detected by confocal imaging in our organoid ptGPC model (Figure 3H), quantified by ImageJ by measuring mean fluorescence intensity of different images and independently confirmed in an additional wt/ptGPC pair of lines (Figures 3I and S3A). Results were in agreement with the RNA sequencing data, since vimentin was one of the top downregulated genes as visualized in the heatmap (Figure 3C).

Considering the significant changes in genes regulating cytoskeletal organization detected in the transcriptome of ptGPC lines, along with previous studies linking KRT17 to progenitor-like populations and epithelial plasticity of the fallopian tube and ovarian cancer,^17^^,^^18^^,^^19^^,^^20^ we investigated whether KRT17 has a functional role in the development of platinum resistance. Though initially below the (p-adjust) threshold in bulk RNA profiling, transcriptional upregulation in KRT17 organoids in response to carboplatin and subsequent ODR-testing was confirmed by qPCR in independent control versus ptGPC1 paired samples of four different donors (Figure 3J). A gradual stepwise increase in KRT17 levels under repeated carboplatin treatment was also validated in control, ptGPC1, and ptGPC2 triplet of organoids by qPCR (Figure S3B). Congruently, a rise in KRT17 levels in ptGPC1 organoids was visualized by confocal imaging (Figure 3K), in two independent pairs of wt/pGPC lines and quantified by ImageJ (Figures S3C and S3D).

### KRT17 overexpression increases the platinum resistance of the organoids

**Figure 4:**
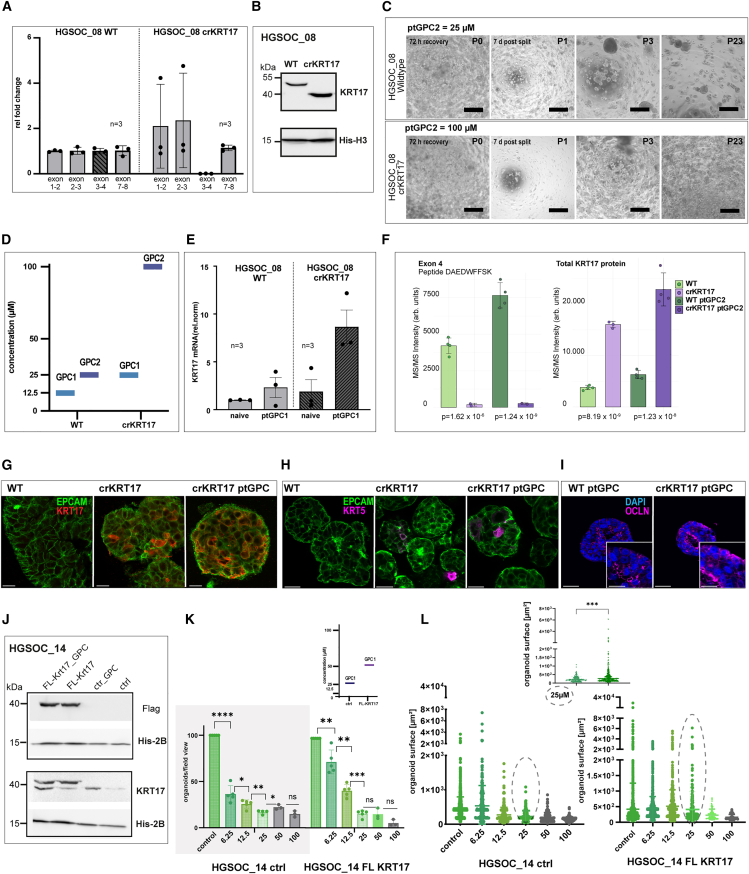
Platinum hyper-resistance is induced by *KRT17* editing (A) qPCR of individual *KRT17* exons, in control vs. crKRT17 edited lines, shows the loss of expression of exon 4 (*n* = 3 independent biological replicates, Error bars, mean ± SEM). (B) Western blot for KRT17 confirms the expression of the truncated protein. (C) Comparative phase contrast images after ODR2-test for WT crKRT17 lines illustrating long-term expansion potential after sustaining 25 μM and 100 μM challenge in P0. Scale bars, 500 μm. (D) Bar plot summarizing the shift in GPC levels and a strong increase in the crKRT17 line (μM). (E) *KRT17* RNA expression by qPCR of treated and untreated, WT and crKRT17 lines, illustrates strong induction in the expression of *KRT17* in crKRT17 organoids (*n* = 3 independent biological replicates). Error bars, mean ± SEM. (F) Mass spectrometry data show a significantly lower or absent MS/MS intensity of the DAEDWFFSK peptide, corresponding to the coding region of exon 4, in edited organoid lines compared to total KRT17 protein intensity (*n* = 4 independent biological samples), confirming the overexpression of the remaining portion of the protein in ptGPC2 organoids. (G) Confocal imaging validates a strong increase in KRT17 levels (red) and (H), KRT5 levels (magenta). Scalebars, 20 μm. (I) Redistribution of Occludin staining signal (magenta) to lateral membranes in crKRT17 ptGPC2 organoids indicates loss of polarity. Scalebar, 50 μm and 25 μm for zoomed-in images (lower right). (J) Western blot for KRT17 expression in FL-KRT17 and control lines, with Flag and endogenous KRT17 antibody, confirms the expression of the exogenous construct as well as the induction of endogenous protein in response to carboplatin. (K) Quantification of the ODR1-test of FL-KRT17 vs. lenti-ctrl organoids showing the increase in organoid counts and rise in GPC. GPC1 = 25 μM (lenti-ctrl) and GPC1 = 50 μM (FL-KRT17). (L) Analysis of the distribution of organoid sizes reveals a group of significantly larger organoids (dotted circles) in ptGPC FL KRT17 culture, suggestive of increased progenitor potential. Error bars represent mean ± SEM of measurements in independent wells. P-values are calculated by the Student’s t test for the quantification of organoid counts, and the Mann-Whitney U test for comparison of differences in organoid sizes (∗*p* < 0.05, ∗∗*p* < 0.01; ∗∗∗*p* < 0.001, ∗∗∗∗*p* < 0.001).

To assess the functional relevance of KRT17 in mediating an increase in resistance, we edited the *KRT17* gene locus using CRISPR/Cas9, introducing specific gRNAs by nucleofection, followed by the generation of a monoclonal line. Interestingly, instead of abolishing *KRT17* expression, CRISPR/Cas9 editing resulted in an efficient in-frame excision of 96 bases in exon 4, as confirmed by Sanger sequencing (Figures S4A–S4C), suggesting a strong selective advantage for this outcome. qPCR analysis of individual *KRT17* exons in the crKRT17 line showed complete loss of exon 4 expression, while the transcription of all other tested exons remained unchanged, indicating that the in-frame deletion did not effectively disrupt overall gene expression (Figure 4A). In alignment, Western Blot revealed a truncated KRT17 protein at ∼40 kDa, with the absence of a full-length protein in the CRISPR/Cas9-engineered donor line (Figure 4B). We then investigated whether the truncation of the KRT17 protein influences the response of organoids to carboplatin by ODR testing. Compared to the WT ptGPC lines, crKRT17 ptGPC lines exhibited strongly increased resistance to consecutive carboplatin treatment (two rounds of ODR-testing), as organoids regrew in P1 following the exposure to maximal (100 μM) carboplatin concentration (Figures 4C, S3D, and S3E). Consequently, GPC1 and GPC2 levels were higher in the crKRT17 line (GPC1 25 μM, GPC2 100 μM), when compared to the WT line (GPC1 12.5 μM, GPC2 25 μM) (Figure 4D). Notably, the quantification of the KRT17 gene expression level in crKRT17 ptGPC2 organoids compared to WT controls detected a strong upregulation in the transcripts from remaining exons following repeated carboplatin treatment (Figure 4E), suggesting the existence of synergistic mechanisms between the effect of carboplatin and regulation of the *KRT17* gene locus. To confirm the upregulation of KRT17 at the protein level and specific truncation at the peptide level, we performed a DIA-based proteomics experiment. The results showed an overall increase in total KRT17 intensity in crKRT17 non-treated and ptGPC2 lines in comparison to WT counterparts (Figure 4F), which fully aligned with qPCR data. Notably, we found one peptide, DAEDWFFSK, that corresponds to the genomic deletion in exon 4, which has significantly lower or absent MS/MS intensity in the edited line (Figure 4F) (*p*-value = 1.6 × 10^−6^ and *p*-value = 1.24 × 10^−9^ for control and ptGPC lines, respectively). At the same time, platinum treatment further strongly boosted levels of all other KRT17 peptides in the crKRT17 lines (total protein) (*p*-value = 8.19 × 10^−9^ and *p* = 1.23 × 10^−8^), and upregulation was confirmed for all individual exons (Figure S4F). An additional strong increase in KRT17 levels, triggered by the edit, was also confirmed by confocal imaging (Figure 4G). Further, hyper-resistant crKRT17 ptGPC2 organoids exhibited an increase in KRT5 expression, a mediator of dedifferentiation and marker of epithelial stages of increased plasticity,^45^ as well as in the distribution of the occludin staining to basolateral membranes, indicating further loss of polarity (Figures 4H and 4I). The precise mechanistic link between KRT17 and KRT5, however, remains to be elucidated.

Despite the conclusive data and penetrant phenotype observed in crKRT17 organoids, confirming that increased KRT17 expression drives platinum resistance, we recognized that the truncated version of KRT17 may have acquired functional properties not present in the native full-length protein. To directly test the ability of KRT17 to modulate carboplatin response, we therefore adopted a complementary and alternative experimental strategy. Full-length (Myc-DDK tagged) *KRT17* was introduced by lentiviral transduction into a different PDO line (HGSOC_14) to additionally exclude donor-specific effects (Figure S5A). The protein expression from the full-length (FL) *KRT17* open reading frame was validated by both Flag antibody detecting the tagged protein as well as with KRT17 antibody detecting both endogenous and slightly larger Flag-Myc tagged KRT17 protein (double band) (Figure 4J). ODR-testing revealed that the expression of the exogenous KRT17 leads to a strong increase in the organoid count, in comparison to the organoids transduced with the control vector (Figure 4K). In agreement with this, GPC1 shifted from 25 μM to 50 μM (Figure 4K), confirming the ability of KRT17 to directly increase platinum resistance. Interestingly, carboplatin treatment in the range 12.5–25 μM led to the appearance of significantly bigger, larger organoids in Flag KRT17 ptGPC1 lines in P1 (Figure 4L), which subsequently translated into a strong increase in the growth rate of the FL-KRT17 organoids during subsequent passaging, quantified by splitting ratio (Figure S5C).

### K-score is an independent negative prognostic factor of OS and PFS in patients with high-grade serous ovarian cancer

To investigate the association of KRT17 expression with clinical outcomes, we have conducted an exploratory analysis of clinical patient data to quantify the expression frequency of KRT17 across a high number of patients with HGSOC and to evaluate its prognostic impact. Therefore, tissue-microarrays (TMAs) of 384 patients diagnosed with primary HGOSC at advanced disease stages (FIGO III-IV) were stained for KRT17 by immunohistochemistry. Clinical characteristics and descriptive statistics of the patient cohort are listed in Tables S4 and S5.

**Figure 5:**
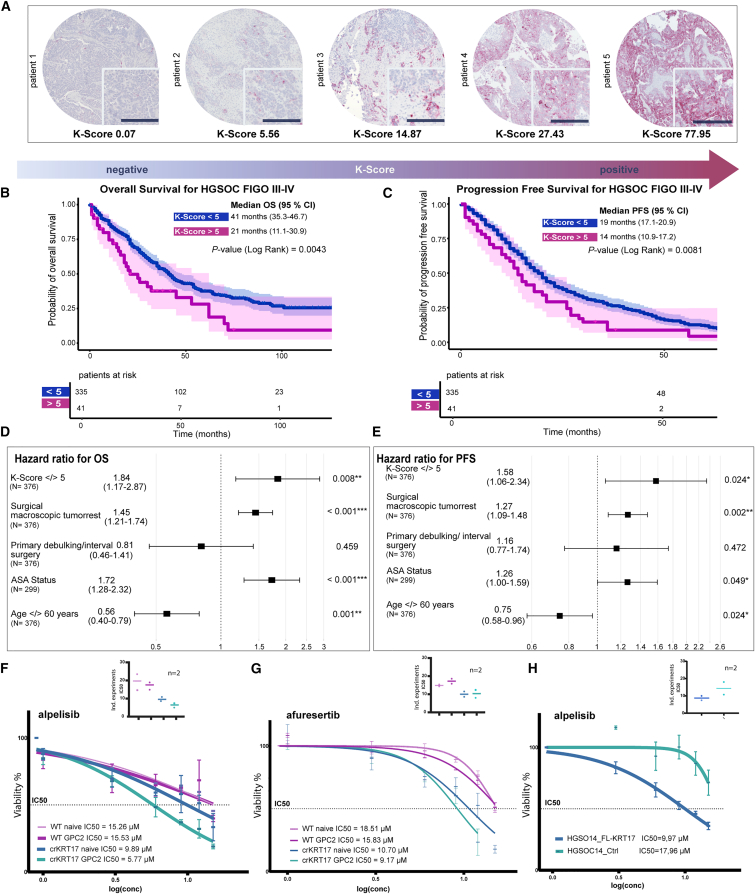
Prognostic K-score as a stratification for PIK3/Akt-targeted therapy (A) Images of KRT17 immunohistochemical staining representing TMA cores of 5 exemplary patients displaying different levels of expression. Scale bar 200 µm. (B) Kaplan-Meier analysis in the patient cohort of advanced HGSOC cases (*n* = 376). K-score cutoff 5 reveals significantly shorter OS (*p*-value (Log Rank) = 0.0043) and (C), PFS (*p*-value (Log Rank) = 0.008) for the group of patients with KRT17^high^. (D) Multivariate Cox regression analysis for OS and (E), for PFS, confirms the prognostic value of the K-score as an independent risk factor (HR 1.84, *p*-value = 0.008 for OS, and HR 1.58, *p*-value = 0.024 for PFS). (F) Log-transformed dose-response curves for alpelisib in untreated and carboplatin-treated (ODR test) WT and crKRT17 lines show a strong increase in sensitivity in the lines expressing elevated KRT17 levels. Upper right panel: IC50 values (μM), obtained from two (*n* = 2) independent repetitions of the assay performed with the same organoid lines. (G) Drug response curves and overview of IC50s of the analogous testing setup for afuresertib. (H) Alpelisib treatment of the FL-KRT17 expressing HGSOC_14 line confirms the capacity of KRT17 to directly modify the sensitivity of the organoids to PI3 kinase pathway inhibition, and an overview of IC50s of independent repetitions of the assay (upper right). Error bars represent mean ± SEM of technical triplicates.

The evaluation of the KRT17 expression was conducted by digital AI-based imaging analysis^38^ (Figure S5D). To quantify expression levels, we developed the K-score as a measure of the KRT17 expression, which is based on the number of positively stained tumor cells in proportion to the overall detected tumor cells and ranged between 0 and 200 (Figure 5A; Table S6). The K-score provides a relative, semi-quantitative measure of KRT17 expression across samples. The average KRT17 expression across all tested tumors was generally low, with a mean K-score of 2.82 (Figure S5E). By using the biomarker cutoff finder tool,^46^ we defined a K-score of 5 as a benchmark to separate the populations of KRT17^high^ and KRT17^low^ tumors. In Kaplan-Meier-analysis, the median overall survival for patients with KRT17^high^ was 21 months (95% CI 35.3–46.7 months) compared to 41 months (95% CI 11.1–30.9 months) for patients with KRT17^low^ (*p* = 0.0043) (Figure 5B). Median progression-free survival for patients with KRT17^high^ was 14 months (95% CI 10.9–17.2 months) compared to 19 months (95% CI 17.1–20.9 months) for patients with KRT17^low^ (*p* = 0.0081) (Figure 5C). Cox regression multivariate analysis, controlled for the main factors influencing the prognosis (general health status of the patients (ASA), age at first diagnosis, postoperative residual disease and neoadjuvant chemotherapy) showed that the K-score is an independent prognostic factor on overall survival with a Hazard ratio of 1.84 (CI 1.17–2.87) (Figure 5D) and progression free survival with a Hazard ratio of 1.58 (CI 1.06–2.34) (Figure 5E). Taken together, the data consistently showed that KRT17 not only has a functional role in the cellular response to platinum in organoids, but that its expression level in tumor tissue has prognostic significance in a large cohort of patients with advanced HGSOC, thus, making it a possible therapeutic target.

### KRT17 is a potential stratification marker for PI3K/Akt-pathway-targeted therapies

KRT17’s involvement in cellular stress response by the activation of downstream signaling, including the PI3K/Akt pathway, has previously been reported in ovarian cancer models.^23^^,^^37^ By now, the PI3K-inhibitor alpelisib and pan-Akt-inhibitor afuresertib have shown tolerable safety profiles for the treatment of HGSOC at the recurrent platinum-resistant stage, but failed to reach primary endpoints in studies designed to include all-comer study populations.^47^^,^^48^^,^^49^ We tested whether a high KRT17 expression might be a potential stratification factor to determine which patients might benefit from PIK3-Kinase- and/or Akt-inhibition. Therefore, we exposed four organoid lines with the same genetic background, differing only at the edited *KRT17* locus (WT naive PDOs, WT ptGPC2, crKRT17 naive, and crKRT17 ptGPC2), to alpelisib and afuresertib. Two of these lines (WT ptGPC2 and crKRT17 ptGPC2) had previously undergone platinum exposure by consecutive ODR testing to model an increase in resistance. This models a sequential therapy strategy of platinum-resistant cancer to targeted therapy.

CrKRT17 non-treated control PDOs (naive), overexpressing KRT17, were found to be more sensitive to alpelisib (IC50 9.89 μM vs. controls IC50 15.26), and the crKRT17 ptGPC2 PDOs showed the lowest IC50 value of 5.77 μM, suggesting the additive effect of platinum treatment and crKRT17 edit, an effect which was stable and reproducible in independent set of experiments (IC50 plot) (Figure 5F). In the case of afuresertib, high levels of KRT17 induced sensitivity already in the crKRT17 non-treated control PDO line (IC50 10.70 μM), without further reduction in the crKRT17 ptGPC2 line (IC50 9.17 μM, Figure 5G). Finally, we confirmed the role of KRT17 in modulating organoid response to alpelisib, with results from the FL-KRT17 line, revealing a pronounced increase in sensitivity compared with control-transduced organoids (Figure 5H). These results suggest that rising KRT17 levels, together with previous exposure to chemotherapy, were associated with increased sensitivity of the examined PDO models to PI3K/Akt pathway inhibition. While the inhibition of PI3K signaling by alpelisib appeared to be more effective in carboplatin-pretreated organoids, pan-Akt inhibition by afuresertib primarily targets the KRT17 status irrespective of the carboplatin effect. In conclusion, the KRT17 expression may influence responsiveness to PI3K/Akt inhibitors, providing a hypothesis-generating basis for the further validation of the K-score in clinical cohorts of advanced and platinum-treated HGSOC.

## Discussion

In the present study, we developed the ODR-test as an experimental approach to quantify platinum resistance in ovarian cancer and study cellular plasticity, which is driven by the response to therapy. Although different groups previously reported an association between drug resistance and enrichment of stemness-related phenotypes within tumors, the mechanism of how chemotherapy resistance emerges remains poorly understood.^15^^,^^50^^,^^51^ Since organoid growth in culture is based on the preservation of epithelial progenitor potential, the ODR-test determines and quantifies the conditions under which individual tumors can sustain regeneration and growth capacity despite the damaging effects of carboplatin. This represents an important conceptual advancement to commonly used drug assays, as they only determine the acute and direct toxic effects of the drugs on remaining viable cells.^13^^,^^52^ Clinical experience has long suggested that every ovarian cancer patient develops molecular resistance to repeated platinum treatment over time, even though the extent of resistance and the speed of the process may vary between patients, and could not be translated into a clinically applicable biomarker stratification so far.^5^ Importantly, the ODR-test effectively confirmed the existence of this phenomenon *in vitro*, as organoids consistently exhibited an increase in resistance upon re-challenge, measured by a shift in the organoid count and an increase in GPC, regardless of their clinical outcome. The data is also fully consistent with the hypothesis that carboplatin treatment triggers a conserved mechanism of the cellular response, while the dynamics of the resistance acquisition have a strong individual component that can be analyzed in detail in patient-specific lines. Different degrees of resistance, observed in organoids after repeated ODR testing, also explain why an increase does not carry clinical consequences in all cases, as the basal level of sensitivity of each tumor is likely the result of complex genetic and environmental factors, and good responders remain more sensitive despite a molecular drift toward a resistant phenotype.

Consequently, the ODR test could be a useful approach for the future development of clinical tools to successfully identify patients at high risk of resistance development.^4^ The assay demonstrated robust reproducibility, with GPCs independently confirmed through repeat experiments by different investigators; however, further refinement of the quantification strategy will be required to resolve smaller differences in the sensitivity profiles of organoids and to more accurately assess the individual risk of resistance development in a clinical context. Global analysis of gene expression and the proteome of ptGPC lines revealed changes in expression in cellular networks regulating cell adhesion, cell-cell communication, as well as cytoskeletal components (Figure 3A–3G). The similarities in response of different donor lines of variable basal resistance to carboplatin challenge are evidence of conserved reprogramming mechanisms and cellular plasticity under therapeutic pressure. While more research is needed to fully understand the mechanisms of progenitor potential preservation under platinum treatment, the stable expansion of ptGPC lines offers a valuable model to study this phenomenon. The identification of KRT17 upregulation under carboplatin treatment is congruent with the findings of a previous study showing that KRT17 is responsive to cytotoxic stressors such as platinum-induced DNA damage.^53^ Beyond its structural role in epithelial integrity, KRT17 regulates cell cycle progression, cytoskeletal remodeling, and PI3K/Akt activation. In our model, carboplatin-induced KRT17 upregulation sustained organoid renewal potential, linking therapeutic stress to adaptive reprogramming and resistance. The strong increase in platinum resistance profile that we detected in lines expressing elevated levels of KRT17 by either CRISPR/Cas9 editing of exon 4 or through the genomic integration of additional copies of the *KRT17* gene suggests the ability of KRT17 to directly influence the response to carboplatin and represents evidence of a potential regulatory loop. While our truncated KRT17 line served as a gain-of-function model, it did not represent a naturally occurring phenotype, as no truncations of KRT17 have been reported in the literature. To address this limitation and specifically investigate the role of the intact KRT17 protein, we established a complementary model by overexpressing full-length KRT17 through lentiviral transduction. Altogether, our findings support the hypothesis that platinum-caused DNA damage induces KRT17, which subsequently actively participates in changes in the cytoskeletal organization to enhance non-canonical epithelial differentiation, promoting tumor survival.^23^^,^^34^^,^^37^ This is in line with Moorman et al.,^54^ who demonstrated that aberrant differentiation can drive resistance and poor survival in another epithelial cancer context. The clinical prognostic relevance of KRT17 expression was established by immunohistochemical analysis of the KRT17 expression profile in a large cohort of 384 patients with advanced HGSOC. We found that the KRT17 expression level classified by the immunoreactive K-score has a significant prognostic value for both overall and progression-free survival. The observed prognostic relevance, confirmed by the Cox proportional hazards model and supported by the *in vitro* results on organoids, provides a rationale for further studies and raises the hypothesis that the K-score could serve as a biomarker candidate in a clinical context to determine which patients are likely to develop a platinum-resistant recurrent disease. Since high KRT17 expression has been reported to have negative prognostic significance also in other epithelial cancer entities, our study could be of interest in a broader oncological context.^24^^,^^25^^,^^26^^,^^27^^,^^28^^,^^29^^,^^30^^,^^31^^,^^32^^,^^33^^,^^34^

Genetically modified organoids expressing high levels of KRT17 (ptGPC crKRT17 and FL KRT17 lines), which also displayed high levels of platinum resistance, provided a model to explore the response of ovarian cancer to targeted agents, as they reflect the potentially relevant clinical scenario of heavily pre-treated, platinum-resistant, and highly KRT17-expressing tumors. In these organoids (Figures 5F–5H), we observed an enhanced sensitivity to the pan-Akt inhibitor afuresertib and PI3K inhibitor alpelisib. These findings indicate a potential link between KRT17 expression and sensitivity of organoids to Akt/PI3 kinase inhibition, and suggest that KRT17 merits further investigation as a candidate biomarker and stratification tool in future therapeutic studies targeting Akt/PI3-kinase in the treatment of platinum-resistant, recurrent ovarian cancer.

### Limitations of the study

Patient-derived organoids effectively capture the basic organization of the epithelial compartment of the tumor tissue *in vitro*, as they expand in culture based on the balance of stemness and differentiation mechanisms governing tumor growth. Though principal findings of the ODR testing and analysis of ptGPC lines suggest that the model has sufficient complexity to phenocopy gradual rise in platinum resistance observed in patients, the absence of immunosurveillance and tumor microenvironment likely limits the predictive potential of the model regarding the timing of the disease recurrence, and exact timing when the tumor will reach a resistance level which would be consequential for clinical decision making. In line with this, while K-score is significantly associated with negative patient outcomes, it does not directly identify platinum-resistant patients but is rather an indicator of a faster process of developing resistance during disease progression. Nevertheless, our approach provides a broadly applicable framework for studying chemotherapy adaptation in patient-derived organoids.

## Resource availability

### Lead contact

Requests for resources, reagents, or additional information should be addressed to the lead contact, Dr. Mirjana Kessler (mirjana.kessler@med.uni-muenchen.de).

### Materials availability

Distribution of organoid lines is subject to the approval of the Ethics Committee of LMU and compliance with Data protection regulations. All other materials are available upon reasonable request.

### Data and code availability

Processed annotated feature counts of the RNA-seq data from WT and ptGPC organoids have been deposited in the GEO database under GSE288422 and are publicly available as of the date of publication. The MS proteomics data have been deposited in the ProteomeXchange Consortium via the PRIDE partner repository with the dataset identifier PXD060376 and are publicly available as of the date of publication. No original code has been created for data analysis in this study.

## STAR★Methods

### Key resources table

**Table:** 

REAGENT or RESOURCE	SOURCE	IDENTIFIER
**Antibodies**		

EpCam	R&D-Systems	Cat#Af960; RRID: AB_355745
gamma-H2A.X	Abcam	Cat#ab81299; RRID: AB_1640564
pBRCA1	Abcam	Cat#ab47325; RRID: AB_868469
E-Cadherin	BD	Cat#610181; RRID: AB_397580
Fibrillarin	Invitrogen	Cat#MA3-16771; RRID: AB_2105791
KRT5	Abcam	Cat#ab52635; RRID: AB_869890
KRT17	Abcam	Cat#ab233912
KRT17	Sigma	Cat# HPA045062; RRID: AB_2679204
KRT17	Cell Signaling Technology	Cat#4543; RRID: AB_2133014
TP53	Santa Cruz	Cat#sc-126; RRID: AB_628082
PAX8	Proteintech	Cat#10336-1-AP; RRID: AB_2236705
Vimentin	Abcam	Cat#ab92547; RRID:AB_10562134
Occludin	Invitrogen	Cat#33-1500; RRID: AB_87033
Phalloidin AF488	Inivtrogen/Thermo	Cat#A12379
Alexa Fluor 488 donkey-anti-goat IgG (H+L)	Invitrogen/Thermo	Cat#A32814; RRID: AB_2866497
Alexa Fluor Plus 555 donkey-anti-mouse IgG (H+L)	Invitrogen/Thermo	Cat#A32773; RRID: AB_2762848
Alexa Fluor Plus 647 donkey-anti-rabbit IgG (H+L)	Invitrogen/Thermo	Cat#A32795; RRID: AB_2866496
Cy™5 AffiniPure™ Donkey Anti-Mouse IgG (H+L)	Jackson Immuno Research	Cat#715-175-151; RRID: AB_2340820
DYKDDDDK tag	Proteintech	Cat#66008-4-lg; RRID: AB_2918475
Histone H2B	Abcam	Cat#ab 1790; RRID: AB_302612
Histone H3	Cell Signaling	Cat#4499S; RRID: AB_10544537
HRP Goat anti-Mouse IgG (H+L)	Thermofisher	Cat# G-21040; RRID: AB_2536527
HRP Goat anti-Rabbit IgG (H+L)	Thermofisher	Cat# G-21234; RRID: AB_2536530

**Bacterial and viral strains**		

Cytokeratin 17 (KRT17) Human Tagged Lenti ORF Clone	OriGene	Cat#RC201619L1V
pLenti-C-Myc-DDK Lentiviral Gene Expression Vector	Origene	Cat#SKU PS100064

**Biological samples**		

Tumor microarray (TMA)	LMU University	N/A

**Chemicals, peptides, and recombinant proteins**		

Bovine serum albumin	Biomol	Cat# 01400.1
B-27™ Supplement (50x)	GibcoTM, Thermo Scientific™	17504-044
Fetal Bovine Serum	GibcoTM, Thermo Scientific™	Cat# 10270106
Freezing Medium Cryo-SFM	PromoCell – Human Centered Science®	Cat#C-29910
GlutaMax	GibcoTM, Thermo Scientific™	Cat#35050-038
Glycin (75,06 g/mol)	Sigma-Aldrich®, Merck	Cat#56-40-6
HEPES (1 M)	GibcoTM, Thermo Scientific™	Cat#15630-056
Human FGF10	Peprotech	Cat# 100-26
Human recombinant BMP2	GibcoTM, Thermo Scientific™	Cat# PHC7146
Human recombinant EGF	GibcoTM, Thermo Scientific™	Cat# PHG0311L
Human recombinant Heregulin beta-1	Peprotech	Cat# 100-03
Milk powder, 500 g	Carl Roth	Cat#68514-61-4
Mowiol® 4-88 Water soluble Mounting Medium	Carl Roth	Cat#81381
N-2 Supplement (100X)	GibcoTM, Thermo Scientific™	Cat# 17502-048
Nicotinamide (NIC) (NO636)	Sigma-Aldrich®, Merck	Cat#98-92-0
N-Acetylcysteine (NAC)	Sigma-Aldrich®, Merck	Cat# A9165-5G
N, N, N, N′-Tetramethylethylendiamin	Sigma-Aldrich®, Merck	Cat#T9281
pEGFP-1 Vector	BD Biosciences	Cat# 6086-1
PageRuler Prestained Protein Ladder	Thermo Scientific™	Cat#26616
Ponceau S solution	Santa Cruz Biotechnology	Cat#6226-79-5
Opti-MEMTM	GibcoTM, Thermo Scientific™	Cat#31985070
ROCK1 and 2 inhibitor Y-27632	TOCRIS biotechne®	Cat#1254
Rspondin-1	By our laboratory	N/A
SYBR Safe DNA Gel Stain	Invitrogen, Thermo Scientific™	Cat#S33102
TaqMan® Universal PCR Master Mix	Applied Biosystems™, Thermo Scientific™	Cat#1258699
Triton® X-100	Sigma-Aldrich®, Merck	Cat#9036-19-5
Trizma® Hydrochlorid	Sigma-Aldrich®, Merck	Cat#1185-53-1
Trypan Blue Stain	Sigma-Aldrich®, Merck	Cat#T10282
TrypLE™ Express Enzyme	GibcoTM, Thermo Scientific™	Cat#12605-028
Tween 20	Carl Roth	Cat#9005-64-5
Zeocin™	GibcoTM, Thermo Scientific™	Cat#R250-01
293T HA Rspo1-Fc	R&D systems	Cat#3710-001-01
HEPES (1 M)	GibcoTM, Thermo Scientific™	Cat# 15630-056
A-83-01 (TGF-β RI Kinase inhibitor IV)	Merck	Cat#616454
Advanced DMEM/F-12 Medium	GibcoTM, Thermo Scientific™	Cat#12634
AmpliTaq DNA polymerase	Thermo Scientific™	Cat#N8080161
Cultrex® Reduced Growth Factor (RGF) Basement Membrane Extract Type II (BME2), Pathclear	R&D systems	Cat#3432-010-01
Cytiva Amersham™ ECL™ Prime Western-Blot-Detection Reagent	Thermo Scientific™	Cat#12994780
DAB+ Substrate Chromogen System (Dako Omnis)	Agilent	Cat#GV82511-2
DAKO Citrate Buffer, pH 6.0, 10x Antigen Retriever	Sigma-Aldrich®, Merck	Cat#C9999-1000ML
Dodecylsulfate-Na-salt in pellets	SERVA	Cat#180055
Epredia™ Richard-Allan Scientific™ HistoGel™	Thermo Scientific™	Cat#12006679
Carboplatin Kabi 600 mg/60 ml	Fresenius Kabi GmbH	Cat# 877418
Afuresertib (GSK2110183)	Selleck Biotechnology	Cat# 1047644-62-1
Alpelisib (BYL719)	Selleck Biotechnology	Cat# 1217586-61-7

**Critical commercial assays**		

CellTiter-Glo® 3D Cell Viability Assay	Promega	Cat #G9681
KRT17 Exon1-2	Thermo Scientific™	Hs01588578_m1
KRT17 Exon2-3	Thermo Scientific™	Hs01588579_gH
KRT17 Exon3-4	Thermo Scientific™	Hs01588580_g1
KRT17 Exon7-8	Thermo Scientific™	Hs00356958_m1
ID2	Thermo Scientific™	Hs04187239_m1
ID3	Thermo Scientific™	Hs00171409_m1
LEF1	Thermo Scientific™	Hs01547250_m1
FOXM1	Thermo Scientific™	Hs01073586_m1
PAX8	Thermo Scientific™	Hs00247586_m1
PROM1 (CD133)	Thermo Scientific™	Hs01009259_m1

**Deposited data**		

RNA Seq data are deposited in the GEO database under GSE288422		
MS proteomics data can be accessed at the ProteomeXchange Consortium with identifier PXD060376		

**Experimental models: Cell lines**		

Patient-derived organoidlines: HGSOC_06, HGSOC_08, HGSOC_14, HGSOC_20	Department for Gynecology and Obstetrics, LMU Hospital, Munich, Germany	N/A

**Oligonucleotides**		

KRT17, CAGCCCCUAAGAGAGCCCUC, ACUGGUCACGCAUCUCGUUG	Synthego, custom design	N/A
TRAC, CUCUCAGCUGGUACACGGCA, GAGAAUCAAAAUCGGUGAAU, ACAAAACUGUGCUAGACAUG	Synthego, custom design	N/A

**Software and algorithms**		

7500 Fast SDS v1.5.1	Applied Biosystems™, Thermo Scientific™	4351104
Fiji ImageJ Software	https://imagej.nih.gov/ij/inde x.html	RRID: SCR_002285
Keyence Viewer and Analyzer Software VR-A2E	KEYENCE GmbH	N/A
LAS X core Software	Leica Microsystems	RRID: SCR_013673
Prism 10.0	GraphPad by Dotmatics	RRID: SCR_002798
QuPath (v0.4.4)	https://qupath.github.io/	RRID: SCR_018257
R Statistical Software (v4.3.2)	R Core Team 2021	RRID: SCR_001905
RStudio packages: dplyr (1.1.4), tidyverse, (2.0.0), ggrepel (0.9.6), Limma (3.60.6), EnhancedVolcano (1.22.0), clusterProfiler (4.12.6), enrichplot (1.24.4), org.Hs.eg.db (‘3.19.1), DESeq2 (1.46.0), pheatmap (1.0.12), clusterProfiler (4.14.4), org.Hs.eg.db (3.20.0), AnnotationDbi (1.68.0), DOSE (4.0.0), enrichplot (1.26.05), ggplot2 (3.5.1)	Posit PBC	RRID:SCR_019186, RRID:SCR_017393, RRID:SCR_010943, RRID:SCR_018931, RRID:SCR_015687, RRID:SCR_016418, RRID:SCR_016418, RRID:SCR_023487
SkanIt RE 7.0.2	Thermo Scientific™	N/A
SPSS Version 29.0.1.0 (171)	IBM SPSS Statistics	RRID: SCR_002865

**Other**		

AllPrep DNA/RNA/protein Kit	QIAGEN	Cat#80204
cDNA Synthesis Kit	Biozym Scientific GmbH	Cat#331470X
Gene knockout kit	Synthego Corporation	N/A
CRISPRevolution Controls Kit	Synthego Corporation	N/A
QIAquick Gel Extraction Kit	QIAGEN	Cat#28704
Qubit™ RNA HS Assay Kit	Thermo Scientific™	Cat#Q32852
RNeasy Mini Kit	QIAGEN	Cat#74104
ZytoChem Plus (HRP) Polymer Kit	BIOZOL Diagnostica Vertrieb GmbH	Cat#ZYT-POLHRP-100
7500 Fast Real-Time PCR System	Applied Biosystems™, Thermo Scientific™	Cat#4351104
Leica TCS SP8 X White Light Laser Confocal	Leica Microsystems	
BZ-X800 Fluorescence microscope	KEYENCE GmbH	RRID: SCR_023617
ChemiDoc MP Imaging System	Bio-Rad Laboratories	Cat#12003154
Countess™ 3FL Automated cell counter	Thermo Scientific™	Cat#AMQAF2000
Gel Doc™ XR+ imaging system	Bio-Rad Laboratories	N/A
Illumina PE150 Novaseq platform	Novogene Co., Ltd.	N/A
Leica TCS SP8 X White Light Laser Confocal Microscope	Leica	N/A
Nalgene® Mr. Frosty	Sigma-Aldrich®, Merck	Cat#5100-0001
NanoDrop® ND-1000 Spectrophotometer	Thermo Scientific™	N/A
NEPA21 electroporator	Nepagene	N/A
Qubit 4 Fluorometer	Thermo Scientific™	Cat#Q33238
Trans-Blot Turbo Transfer System	Bio-Rad Laboratories	N/A
Varioskan™ LUX multimode microplate reader	Thermo Scientific™	Cat#15350747, RRID: SCR_026792
ZytoChem Plus HRP Polymer System (Mouse/Rabbit)	Zytomed	Cat#POLHRP-100

### Experimental model and study participant details

#### Human subjects

The study was conducted following the Ethics Committee of the Ludwig-Maximilian-University (LMU), Munich, Germany (17-0471), and in compliance with all legal EU, national, and local regulations. Each patient provided written informed consent to participate. All subjects were female, and gender was considered irrelevant to the analysis. The average age of the patients at the time of diagnosis was 65.69 years. A detailed overview regarding staging and other key clinical characteristics is provided in the Tables S1, S4, and S5. An ovarian cancer biobank, established following our institutional protocols,^12^^,^^55^ was a source of primary high-grade serous ovarian cancer patient-derived organoid (HGSOC PDO) lines that were used in this study, including embedded tissue samples, along with their clinical data. A routine pathological assessment of the primary tumor tissue confirmed the malignant, non-borderline diagnosis based on standard histomorphology characteristics. The samples were collected during multi-visceral surgeries at the Department for Gynecology and Obstetrics, LMU Hospital, Munich, Germany. Gyneco-oncological surgeons collected clean peritoneal tumor deposits, avoiding macroscopically necrotic areas, and the tissue was processed in the laboratory for organoid generation.^12^^,^^55^ Embedded tissue and corresponding organoid cultures exhibit matching molecular profiles.^8^

Tissue microarrays (TMA) for immunohistochemical analysis were prepared and stained by the LMU Department of Pathology, comprising a total of 384 duplicated samples of HGSOC patients diagnosed with advanced-stage disease (FIGO III-IV) treated at the department. Clinical data were available for further analysis.

### Method details

#### Organoid cultivation

HGSOC PDOs selected from the departmental biobank were cultivated in individually optimized media. Donor lines included in the study all grew in OCM1 medium (low WNT, high BMP environment) with two lines showing benefit from the addition of her 1β (Table S7).^12^ Passaging of organoids, cryopreservation, and thawing procedures followed protocols established by our laboratory.^12^ Briefly, depending on organoid growth, every 10–20 days, organoids were released from Cultrex® BME 2 and trypsinized with TrypLE™ (Gibco™). Depending on the grade of the desired dissociation, the cell suspension was strained through cell filters to ensure singularity. Dissociated cells were reseeded in the matrix, and ovarian cancer medium was added after 30 min of solidification. Cryopreservation of organoids was performed in the proliferation phase of organoid growth in the first week post-passage. Organoids were pelleted and transferred to 1 ml Cryopreservation medium (e.g., Freezing Medium Cryo-SFM, PromoCell – Human Centered Science®) and stored initially at -80°C freezing container (e.g., Nalgene® Mr. Frosty, Sigma-Aldrich®), before transferring to liquid nitrogen for long-term stable storage.

#### *In vitro* cell viability drug assay

Organoids were trypsinized, strained through the filter, counted, and 30,000 cells were seeded in a 48-well plate format in triplicate. At day 5-7 post-seeding, depending on the growth dynamic of the individual line, fully formed organoids were treated with carboplatin at increasing concentrations (dilution in organoid medium to 6.25 μM, 12.5 μM, 25 μM, 50 μM, and 100 μM), alongside a control. The cell viability was measured after 72 h using the CellTiter-Glo® 3D Cell Viability Assay (Promega) according to the manufacturer’s directions at the Varioskan™ LUX multimode microplate reader (Thermo Scientific™). Drug response for PI3K/Akt inhibitors alpelisib and afuresertib was measured in the same format, with a treatment duration of five days. The individual inhibitory concentration at 50 % cell viability was determined for each tested PDO line, in Graph Prism software, by calculating the dose response by applying a nonlinear regression model.

#### Organoid drug resistance (ODR) test

The regenerative potential of organoids was tested by subjecting them to different doses of carboplatin using our developed ODR test (Figure 1D). Initially, organoids were singularized by trypsinization, mechanical dissociation, and straining through a 200 μm filter for equal distribution and seeded at a density of 50,000 cells/well in a 24-well plate format. At day 7 post-passage, the organoids were treated with carboplatin for 48 h at five increasing concentrations (dilution in organoid medium to 6.25 μM, 12.5 μM, 25 μM, 50 μM, and 100 μM) alongside a control. After a 72-hour recovery phase in a carboplatin-free organoid medium, passaging was performed to a 48-well format into Passage 1 (P1). A ratio of 1:3 to 1:5, depending on the growth dynamics of the organoid line, is recommended, as excessive seeding density limits space and nutrient availability, thereby restricting organoid growth and resulting in smaller organoids. Organoid generation and growth were quantified by imaging each well at 4x and 10x magnifications using a phase contrast microscope, at 3-, 7-, and 10-days post-split, and the Qupath Software. By further passaging of organoids, we identified the highest carboplatin concentration level in P0, after which unlimited long-term expansion of the line was possible - namely the growth permitting concentration (GPC).

#### RNA isolation and quantitative PCR (qRT-PCR)

The RNeasy Mini Kit (QIAGEN) was used for total RNA extraction according to the manufacturer’s protocol. Organoids were released from the extracellular matrix with cold ADF++ and pelleted. After resuspension in Buffer RLT (QIAGEN) supplemented with ß-mercaptoethanol and homogenization, 70% ethanol was added, and samples were processed via RNA purification columns. Quantification of the RNA concentration and purity was performed with the Qubit 4 Fluorometer (Thermo Scientific™) following the instructions of the Qubit™ RNA HS Assay Kit (Thermo Scientific™). For the two-step quantitative reverse transcription PCR (qRT-PCR), the RNA samples were transcribed to cDNA using the cDNA Synthesis Kit (Biozym). TaqMan® Universal PCR Master Mix, TaqMan® Gene Expression Assay targeting the gene of interest (Table S8) was used for measuring quantitative gene expression on the Applied Biosystems™ 7500 Fast Real-Time PCR System. The quantification of expression levels was performed by the ΔΔCt method relative to the expression of the Glyceraldehyde-3-phosphate Dehydrogenase (GAPDH). Change of expression in the test group (e.g., ptGPC organoids) was determined in relation to the average value of the expression of the gene in the control group. The GraphPad Prism 10 (RRID: SCR_002798) software was used for data analysis and graphical representation.

#### CRISPR-Cas9-mediated editing of organoids

CRISPR-Cas9-mediated gene editing for KRT17 was performed with two customized single guide RNAs (sgRNA) (Synthego) (Table S9). Organoids previously singularized by trypsinization and mechanical dissociation were transfected with the Cas9 endonuclease and sgRNA/ribonucleoparticles (RNP) via electroporation in the NEPA21 electroporator (Nepagene). For this purpose, 150,000 singularized organoid cells each were combined in a total of 50 μl reaction mixture consisting of 25 μL reduced serum medium (Opti-MEM™, Gibco™) and 25 μL assembled RNP mixture (1 μl SpCas9 2NLS Nuclease (Synthego) (20 pmol/μl, 6 μl target-specific sgRNAs (30 pmol/μl), and 18 μl Opti-MEM™). Controls included a positive control by transfection with sgRNA targeting the previously validated *TRAC* gene (Human TRAC Positive Control, Synthego), and a negative (mock) control. After electroporation, organoids were resuspended in an growth medium, pelleted, and singularly seeded in Cultrex®.

#### Validation of gene editing by PCR

Three weeks after electroporation, genomic DNA was extracted using the AllPrep DNA/RNA/protein Kit (QIAGEN), and the targeted locus was amplified. Gel electrophoresis on 2 % agarose gels with SYBR Safe DNA Gel Stain (Invitrogen) visualized the products alongside pBR328 Mix I (Carl Roth) as a DNA marker. A purified PCR product was sent for Sanger sequencing to confirm the edit. Monoclonal organoid lines have been generated by singularization of the pooled cultures, clone picking, and expansion of the individual organoid lines.

#### Western blot analysis

For the preparation of the total protein samples, organoids were harvested and washed with cold PBS. The pellet was resuspended in 4x Laemmli buffer (Bio-Rad), volume adjusted with PBS to ensure 1x Laemmli concentration, and lysates were heated for 10 min at 95°C. After SDS-PAGE, proteins were transferred to a 0.2 μM PVDF membrane by semi-dry Trans-Blot Turbo Transfer System (Bio-Rad), followed by blocking in 3 % milk powder and 1.5 % BSA blocking solution. After overnight incubation with the primary antibody at 4°C and 3 washing steps in TBS/Tween 0.05%, the membrane was incubated with the respective secondary HRP-conjugated IgG-antibody (Table S10) for 1 h at room temperature. After washing, proteins were detected by use of the Cytiva Amersham™ ECL™ Prime Western-Blot-Detection Reagent (Thermo Scientific™) on the ChemiDoc MP Imaging System (Bio-Rad). ß-Actin (Sigma, A5441), Histone-2B (ab1790), or Histone-H3 (ab8580) antibodies were used as endogenous control for the normalization of protein loading.

#### Lentiviral delivery of full-length *KRT17*

Ready-to-use lentiviral particles carrying FLAG (DDK)-Myc -tagged full-length *KRT17*, or control vectors (RC201619L1V and PS100064V), have been transduced following the established protocol.^56^ In short, cell culture plates (24-well format) were pre-coated with a 1:1 mixture of Cultrex and ADF medium and left to solidify overnight at 37°C. Organoids were dissociated into single cells and counted to calculate the MOI, defined as the number of viral particles per target cell. An appropriate number of lentiviral *KRT17* particles and a control vector, to achieve MOI 2, were added to the suspension with the addition of polybrene to facilitate the transduction, and the mixture was transferred to pre-coated plates and incubated for 24h at 37°C. The next day, cells were retrieved and washed, and 3D seeding was performed according to the standard organoid cultivation protocol. Successful transduction and protein expression of the tagged KRT17 construct were validated by Western Blotting.

#### mRNA sequencing

Preparation of the library and NGS Sequencing of the RNA samples from carboplatin-treated and non*-*treated ovarian cancer organoids, isolated by mRNA Easy (Qiagen) kit according to the manufacturer’s protocol, was performed by Novogene Co., Ltd., China, on the Illumina PE150 Novaseq platform, fulfilling stringent quality control criteria (Q30 > 95%). The reads were aligned against the index file built from the GRCh38 primary assembly genome (GENCODE release 45) and quantified using the Rsubread package (2.20.0), with the GRCh38 GENCODE v38 GTF file as the reference. Differential gene expression analysis was performed using DESeq2 (version 1.46.0) in the R environment (version 4.4.2). RStudio Packages DESeq2 (1.46.0), ggrepel (0.9.6), dplyr (1.1.4), pheatmap (1.0.12), clusterProfiler (4.14.4), org. Hs. eg, db (3.20.0), AnnotationDbi (1.68.0), DOSE (4.0.0), enrichplot (1.26.05), and ggplot2 (3.5.1) were used for the analysis and visualization of differential gene expression data.

#### Mass spectrometry analysis

Organoids were singularized, and 50,000 cells were seeded per 50 μl droplet in a 3D matrix, with four technical replicate wells per condition (e.g., control and post-ODR). After 10 days, fully grown organoids were collected, pelleted, shock-frozen in liquid N_2_, and stored at -80°C until lysis. All lysates were prepared on the same date to minimize technical variability. Cell pellets were lysed in Eppendorf tubes with 50 μl of boiling lysis buffer (5 % SDS, 100 mM Tris pH 8.5, 5 mM TCEP, and 10 mM CAA). The lysates were incubated at 95°C for 10 min with mixing (500 rpm) and quantified with Pierce BCA Protein Assay Kit (Thermo Scientific). Protein digestion was performed using a Protein Aggregation Capture (PAC)^57^-based digestion MagReSyn Hydroxyl beads (ReSyn Biosciences) on a King Fisher Flex system (Thermo Scientific).

Samples that were mixed with beads were added to the samples at a protein bead ratio of 1:2. For each sample, 200 μl of digestion solution (50 mM triethylammonium bicarbonate) containing Lys-C and Trypsin at an enzyme-to-substrate ratio of 1:500 and 1:250, respectively. Digestion was performed overnight at 37°C. Protease activity was quenched by acidification with TFA to a final volume percentage of 1%.^58^

For proteome experiments, 500 ng of digested peptide was loaded in an Evotip Pure (Evosep) following the manufacturer’s instructions. Samples were analyzed using an Evosep column (PepSep EV-1109, 8 cm – 150 μm - C18 1.5 μm) and a fused silica (EV-1087 - 20 μm) using an EASY-Spray™ source and interfaced with the Orbitrap Astral Mass Spectrometer (Thermo Scientific). In all samples, spray voltage was set to 1.8 kV, funnel RF level at 40, and heated capillary temperature at 275°C. Samples were separated on an Evosep One LC system using the pre-programmed gradient for 60 samples per day (SPD – 21 min gradient). All experiments were acquired using a data-independent acquisition (DIA) method, cycle time of 0.6 seconds, in a range of 480-1080 m/z, and a maximum injection time (IT) of 30 ms.

#### Data analysis: proteomics

Raw files were analyzed in Spectronaut v19 (Biognosys) with a spectral library-free approach (directDIA+) using the human protein reference 232 database (Uniprot 2022 release, 20,588 sequences) and 233 complemented with common contaminants (246 sequences). Carbamidomethylation of cysteine was set as a fixed modification, whereas oxidation of methionine, N-terminal protein acetylation were set as variable modifications. The enzyme/cleavage rule was set to Trypsin/P, the digest type to specific, and a maximum number of two missed cleavages per peptide was allowed. The maximum number of variable modifications per peptide was set to 5, and method evaluation was turned off. The data analysis of proteome data was performed in R version 4.4.0 (R Core Team, 2021), R Foundation for Statistical Computing, with R Studio 2024.4.0.735. The output files were analyzed using RStudio with the following packages: dplyr (1.1.4), tidyverse (2.0.0), ggrepel (0.9.6), Limma (RRID: SCR_010943) (3.60.6), EnhancedVolcano (1.22.0), clusterProfiler (RRID: SCR_016884) (4.12.6), enrichplot (1.24.4), org Hs. eg., db (‘3.19.1).

#### Fixation and embedding of organoids

Fixation of organoids in Epredia™ Richard-Allan Scientific™ HistoGel™ (Thermo Scientific™) for subsequent paraffin embedding, micro-sectioning, and staining procedures followed our institutional protocol.^12^ In summary, organoids were released from the Cultrex® BME 2 matrix by cold ADF++, fixed in 4 % Paraformaldehyde (PFA), and washed twice in PBS. Fixed organoids were gently resuspended in 100 μl of pre-heated HistoGel™, and the solidified HistoGel™ droplet was processed for paraffin embedding and slicing.

#### IHC and IF staining of organoids

Immunohistochemistry was performed by the HRP-DAB method (Zytochem Plus HRP Polymerkit POLHRP-100). Paraffin sections were deparaffinized, rehydrated, and treated with 3 % H_2_O_2_/methanol. Antigen retrieval was performed in Tris buffer (pH 9.0, 95°C, 30 min). After blocking (Reagent 1), slides were incubated with primary antibody (1:50) overnight at 4°C, followed by post-block (Reagent 2), HRP polymer (Reagent 3), and DAB (1 min). Sections were counterstained with hematoxylin, dehydrated, cleared, and mounted for microscopy.

Immunofluorescence staining was performed as previously described.^12^ In short, slides were deparaffinized in Roticlear® (Carl Roth), followed by rehydration in a descending ethanol series, antigen retrieval by heating in a steamer to 100°C with TRIS/EDTA solution, and permeabilization for 15 min in 1 % Triton® X-100 (in PBS) solution (Sigma-Aldrich®). After blocking with 10% serum and primary antibody incubation at 4°C for at least 16 h, slides were stained with secondary IgG antibodies for 2 h at room temperature. DAPI counterstaining (Thermo Scientific™) visualized nuclei. Mowiol® 4-88 Water soluble Mounting Medium (Carl Roth) sealed the slides for imaging on a Leica SP8 confocal microscope. Quantification was performed in ImageJ on independent images acquired under identical settings. Integrated density was measured within defined ROIs, and the signal of the channel of interest was normalized to either DAPI or EpCAM. Stainings containing phalloidin to visualize actin were performed using a whole-mount protocol. Fixed organoids were washed in PBS, then permeabilized and blocked overnight in 5% BSA in PBS containing 0.05% Tween-20 and 0.5% Triton X-100. Next, organoids were incubated with primary antibodies for 16 h, followed by incubation with secondary antibodies for 2 h at room temperature, during which phalloidin was added.

#### Immunohistochemistry of TMA slides

After the removal of paraffin (Roticlear and decreasing ethanol range), slides were incubated in antigen-retrieval solution pH 8,0 (Novocastra RE7116) for 2 x 15 min in the heated Microwave and left to cool down for 20 min at room temperature. Next, samples were blocked for 10 min with Protein Block (Agilent Technologies, X0909). Primary antibody anti-KRT17 (clone SPM560, ab 233912), diluted 1:250, was incubated for 1 h at room temperature. After washing (PH 7,5 TRIS buffer), samples were treated with AP Polymer anti-Mouse (Zytomed Systems, ZUC-077) for 30 min, washed, and Vector Red substrate was added for 20 min (Vector, SK-5100). Excess dye was washed out for 10 min under running water, counterstaining with Hematoxylin (Vector, H-3401) was performed for 10 sec, and the washing step was repeated. Samples we mounted with Aquatex (Merck, 1.08562.0050).

#### Evaluation of TMA staining

The ovarian cancer TMA slides were visualized by the automated Keyence Microscope BZ-X800 at a 20x magnification with automated image stitching. The QuPath Bioimage Analysis Software (RRID: SCR_018257)^38^ was used for IHC analysis of KRT17 expression on TMA slides. An automated script ensured accurate, consistent, and objective IHC scoring (Table S10). Stains were separated by color deconvolution after smoothing. QuPath’s Pixel and Object Classifier algorithms were interactively trained on more than 30 selected heterogeneous training images within the study collective to automatically annotate regions of interest and to distinguish epithelial tumor cells from all other detections. The QuPath’s Positive Cell Detection algorithm was applied for cell segmentation and counting. Intensity thresholds were determined for either negative, weakly positive, or strongly positive cells based on the cell KRT17 optical density sum (negative < 0.09, weakly positive 0.090.17, strongly positive > 0.17, Figure 4D). Automated batch analysis was applied to all 384 duplicated TMA samples, providing data for further statistical analysis.

### Quantification and statistical analysis

For the TMA analysis, the total cell count for tumor and stroma cells and positive cell detection were pooled for both sample duplicates before further statistical analysis. A tailored immunoreactivity score was established. For this purpose, a weakly positive cell count was added to the twofold count of strongly positive cells, then divided by the total tumor cell count and multiplied by 100, resulting in a range of 0 (all tumor cells negative for KRT17) to 200 (all tumor cells strongly positive for KRT17). We named this adapted immunoreactivity score “K-score”. Samples with less than 1150 detected tumor cells in total were excluded from further statistical analysis, based on the required sample size to classify the sample based on a K score of 5 as a cutoff with > 99,99 % confidence. Clinical information, including data about survival and disease progression, was related to the immunohistochemical scoring by descriptive statistical analysis, Kaplan-Meier analysis, and Multivariate Cox regression analysis by the IBM SPSS Statistical Software (version 29.0.1.0 (171)) as well as Survival (version 3.7.0) and survminer (0.5.0) packages in R Data analysis. To determine an optimal cutoff point for patient stratification, we applied the statistical approach described by Budczies et al.^46^

#### Data visualization

Data visualization was performed using GraphPad Prism Software Version 10.1.1. was used to generate concentration-response curve diagrams for organoid counting and mRNA expression, analyze cell viability data, and create graphical representations of the experimental results obtained in Qupath (organoid count and surface area data). R packages survival, surminer, dplyr, foreign, and ggplot2 were used for data processing and generating plots of survival analysis, and HR plots of Cox proportional model analysis.

## Acknowledgments

Research at the Research Laboratory of the Department of Obstetrics and Gynecology is made possible by the institutional support of the LMU University Hospital. M.K., S.M., F.T., A.C.R., and B.C. obtained the support of the 10.13039/501100012353German Cancer Consortium (DKTK). J.R. and A.C.R. received funding from the Bavarian Cancer Research Center, BKZF. J.R. and A.C.R. obtained fellowships from the LMU Medical and Clinician Scientist Program. F.T. received grant funding from 10.13039/501100005972German Cancer Aid DKH, # 70113426 and #70113433. M.K., J.O., I.P., and S.G. had the support of the ERAPERMED2022-141 (10.13039/501100002347BMBF Grant 01KU2302), I.P. and J.V.O. work at the Novo Nordisk Foundation Center for Protein Research (CPR), which is funded in part by a donation from the 10.13039/501100009708Novo Nordisk Foundation (NNF14CC0001).

The authors would like to thank Linpu Yang, Maria Fischer, Andrea Sendelhofert, Freya Kinzinger, Selina Vogl, Martina Rahmeh, Madlen Leinauer, Cornelia Herbst, Sabine Fink, and Simone Hofmann for their help. We are grateful to the staff of BMC Core Facility for Bioimaging, Klein Lab at Dr. von Hauner Children’s Hospital, who provided S2 Lab space, and the Core facility of the Institute of Anatomy, LMU Faculty of Medicine, where organoid samples were embedded.

## Author contributions

Conceptualization, M.K. and J.R.; methodology, J.R., M.K., I.P., and J.V.O.; validation, J.R., J.S., S.H., T.Z., and S.G.; formal analysis, J.R, J.S, I.P., S.H., S.G, T.Z, A.D., J.V.O., and M.K.; investigation, J.R., J.S., S.H., T.Z., I.P, S.G, J.F., A.C.R., A.D., and N.V.; resources, S.M., C.K., F.T., A.B, B.C., F.K., D.M, and T.S.; writing – original draft, J.R. and M.K.; writing – reviewer and editing, J.R., M.K., F.T., S.M., F.K., B.C., D.M., A.B., T.S., C.K., I.P., J.V.O., S.G., and J.S.; visualization, J.R., J.S., S.G., I.P., and M.K.; supervision, M.K.; project administration, M.K.; funding acquisition, M.K., F.T., J.R., A.C.R., and J.V.O.

## Declaration of interests

The authors report the following competing interests: J.R. received speech honoraria from AstraZeneca. S.M. obtained research funding, advisory board, and honorary or travel expenses from AbbVie, AstraZeneca, Clovis, Eisai, GlaxoSmithKline, Hubro, Immunogen, Medac, MSD, Novartis, Nykode, Olympus, PharmaMar, Pfizer, Roche, Seagen, Sensor Kinesis, and Teva. F.T. received research funding, advisory board, and honorary or travel expenses from AbbVie, AstraZeneca, Eisai, GSK, ImmunoGen, MSD, Regeneron, Roche, and SAGA Diagnostics. B.C. received speech/advisory board honoraria from AstraZeneca and MSD. M.K. is listed as an inventor on the patent for ovarian cancer organoid culture. Other authors have no competing interests.

## Supplemental information

Document S1. Figures S1–S5 and Data S1/Methods S1

Table S1. Clinical characteristics of patients corresponding to organoid lines used in this study, related to Figure 1

Table S2. Differentially expressed genes in ptGPC organoid lines, related to Figure 3

Table S3. Differentially regulated proteins in ptGPC organoid lines, related to Figure 3

Table S4. Summary of patient characteristics in the HGSOC TMA cohort, related to Figure 5

Table S5. Detailed clinical characteristics of patients in the HGSOC TMA cohort, related to Figure 5

Table S6. KRT17-positive cell counts across TMA samples, related to Figure 5

Table S7. Composition of organoid growth media, related to STAR Methods

Table S8. TaqMan primers used in this study, related to STAR Methods

Table S9. Sequences of guide RNAs used for CRISPR editing, related to STAR Methods

Table S10. Parameters used for automatic KRT17 cell detection, related to STAR Methods

Video abstract
