# Supplementary material for: Organoid platinum-resistance model identifies KRT17 as a biomarker of targeted therapy in ovarian cancer
Source: iScience. 2025 Nov 10;28(12):113999. doi: 10.1016/j.isci.2025.113999 (PMC12702183; doi:10.1016/j.isci.2025.113999)
Supplement: Document S1. Figures S1–S5 and Data S1/Methods S1 [file mmc1.pdf]

iScience, Volume *28*

Supplemental information

Organoid platinum-resistance model identifies KRT17 as a biomarker of targeted therapy in ovarian cancer

Juliane Reichenbach, Juliana Schmid, Sophia Hierlmayer, Tingyu Zhang, Ilaria Piga, Sophia Geweniger, Jonas Fischer, Aarushi Davesar, Nemanja Vasovic, Anca Chelariu-Raicu, Fabian Kraus, Alexander Burges, Bastian Czogalla, Doris Mayr, Tobias Straub, Christoph Klein, Jesper V. Olsen, Sven Mahner, Fabian Trillsch, and Mirjana Kessler

Supplemental Information

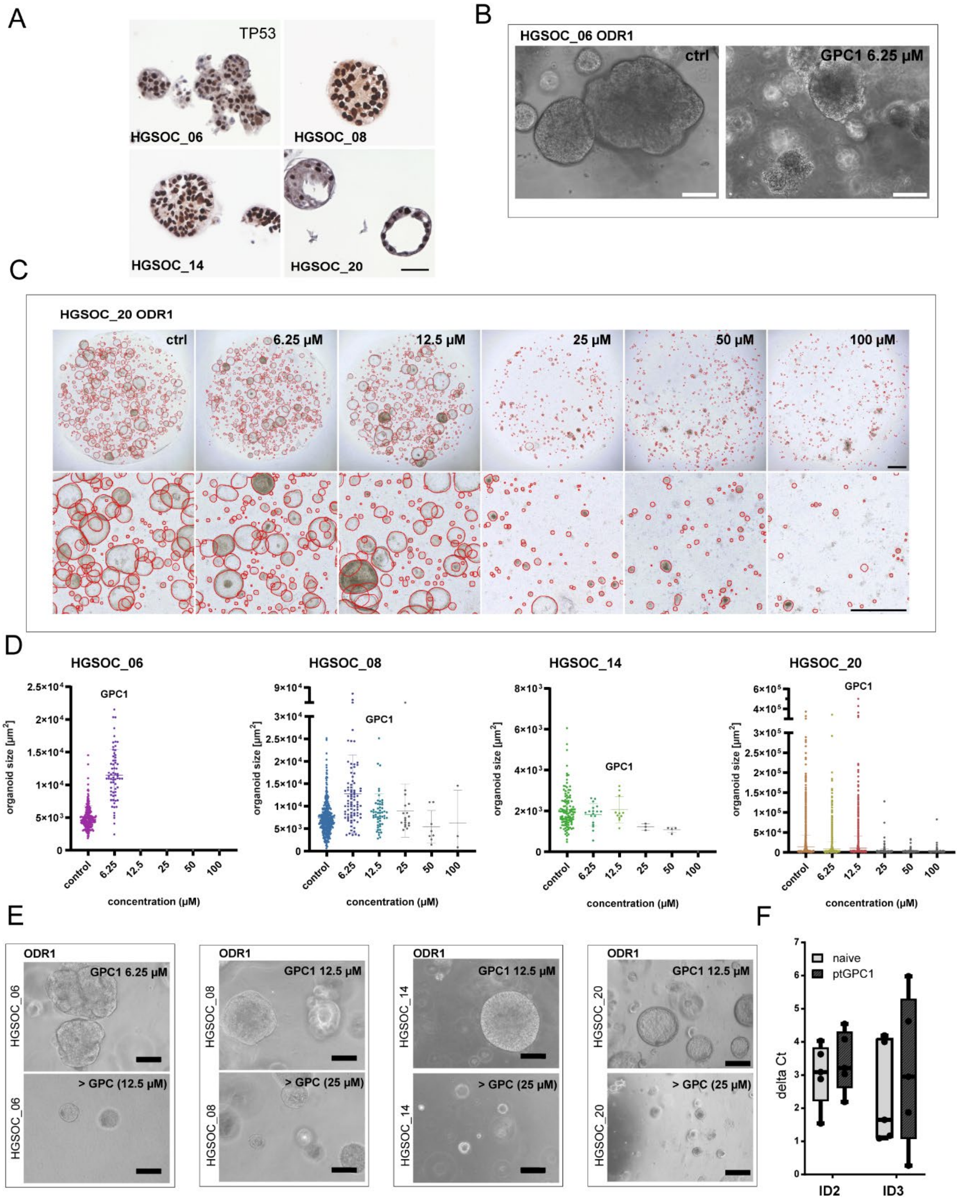

Figure S1

Quantification of the effect of carboplatin on organoid formation and growth, related to Figure 1. (A) Immunohistochemical staining of TP53 in organoid lines demonstrates strong nuclear

overexpression, consistent with a mutant phenotype. Scale bar, 50 µm. (B) Phase contrast exemplary image of the phenotypic changes in organoids at 72 h post-recovery after carboplatin exposure. Scale bar, 100 µm. (C) Illustration of the counting process, and organoid annotation on phase contrast images of a representative PDO line after Carboplatin treatment. (D) A scatter plot depicting the distribution of organoid sizes in μm^2 for each concentration of Carboplatin treatment for four independent organoid lines shows a reduction at higher concentrations. Counting was performed with QuPath-0.5.1. (E) Phase contrast images of a representative line showing differences in organoid size and morphology, and growth permitting concentration vs. higher concentration. Scale bars, 100 µm. (F) Box plots of ΔCt values (normalized to GAPDH) in WT (naïve) and ptGPC organoids show sustained, robust expression of BMP transcription factors ID2 and ID3. Data are presented as mean ±SEM. Samples represent independent pairs of organoid lines, control vs. GPC1 (n = 4).

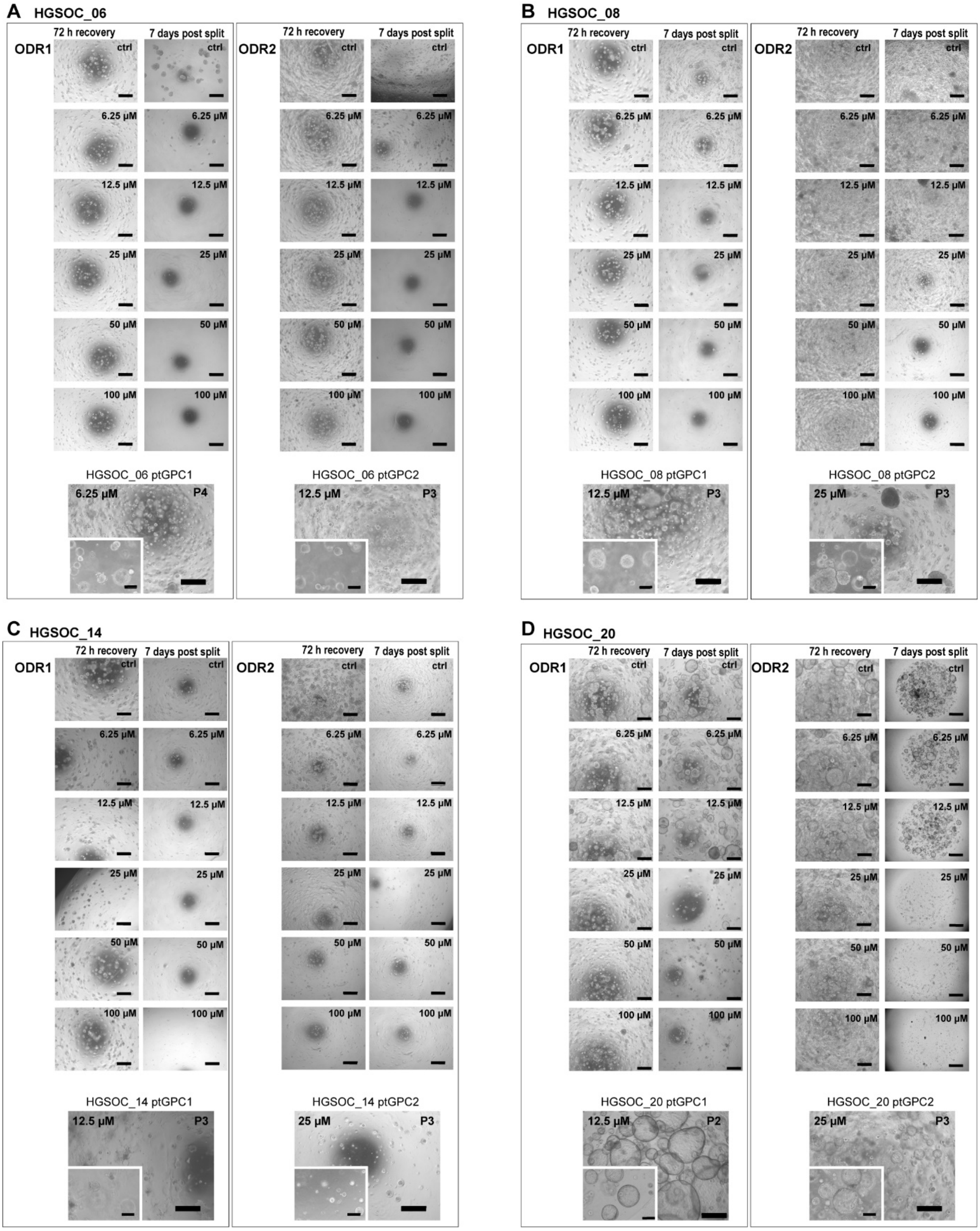

Figure S2

Organoids gain resistance after repeated exposure to carboplatin, related to Figure 2. (A-D) Phase contrast images of three representative lines after 72 h recovery from carboplatin treatment, 7 days post-split (P0), as well as the established line at GPC in stable long-term

cultivation. Comparison of organoid lines' growth after ODR1 and ODR2. Scale bars, 500 and 100 µm (zoom in, lower left).

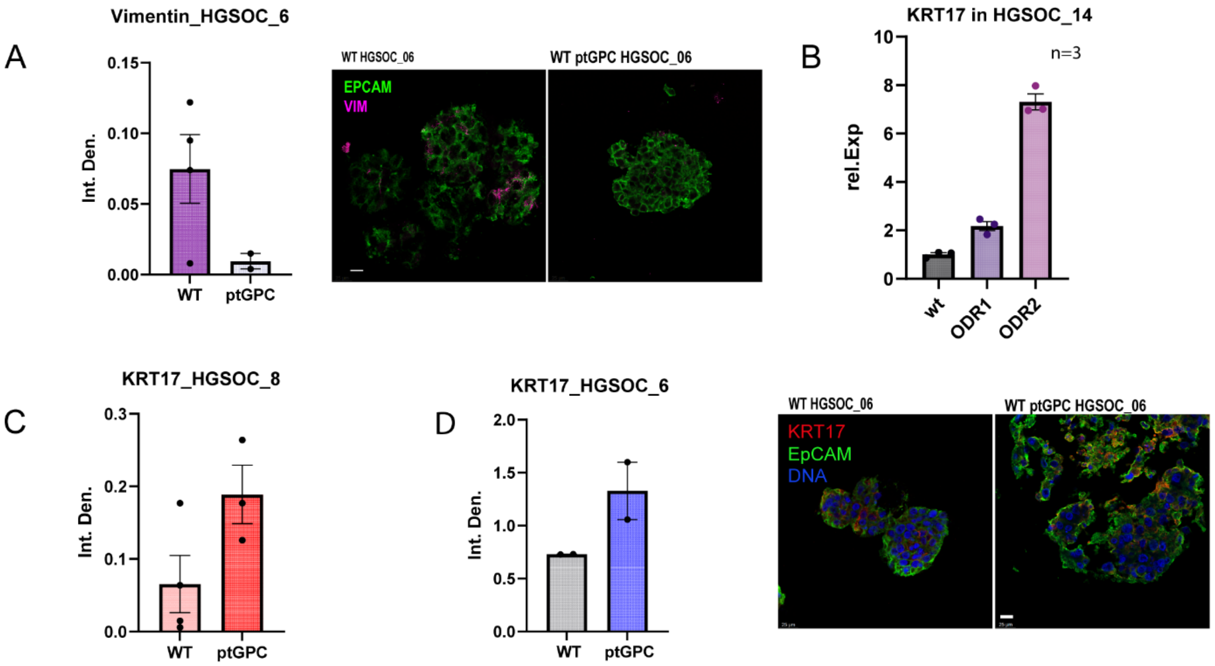

Figure S3

Validation of KRT17 induction and vimentin reduction in post-ODR organoids, related to Figure 3 (H-K). (A) Quantification plot of Vimentin staining in HGSOC_06 WT and ODR1 organoids and a representative IF image. Error bars, mean ±SEM. (B) qPCR of additive induction of KRT17 in control post-ODR1 and ODR2 lines in HGSCOC_14 organoids. N = 3 of three qPCR experiments, data is normalised to the average expression of the WT control group. Error bars, mean ±SEM. (C) Quantification plot of KRT17 expression in HGSOC_08 (related to Fig. 3K). Error bars, mean ±SEM. (D) Quantification plot of KRT17 induction in HGSOC_06 ODR1 line and representative IF Image. Error bars, mean ±SEM.

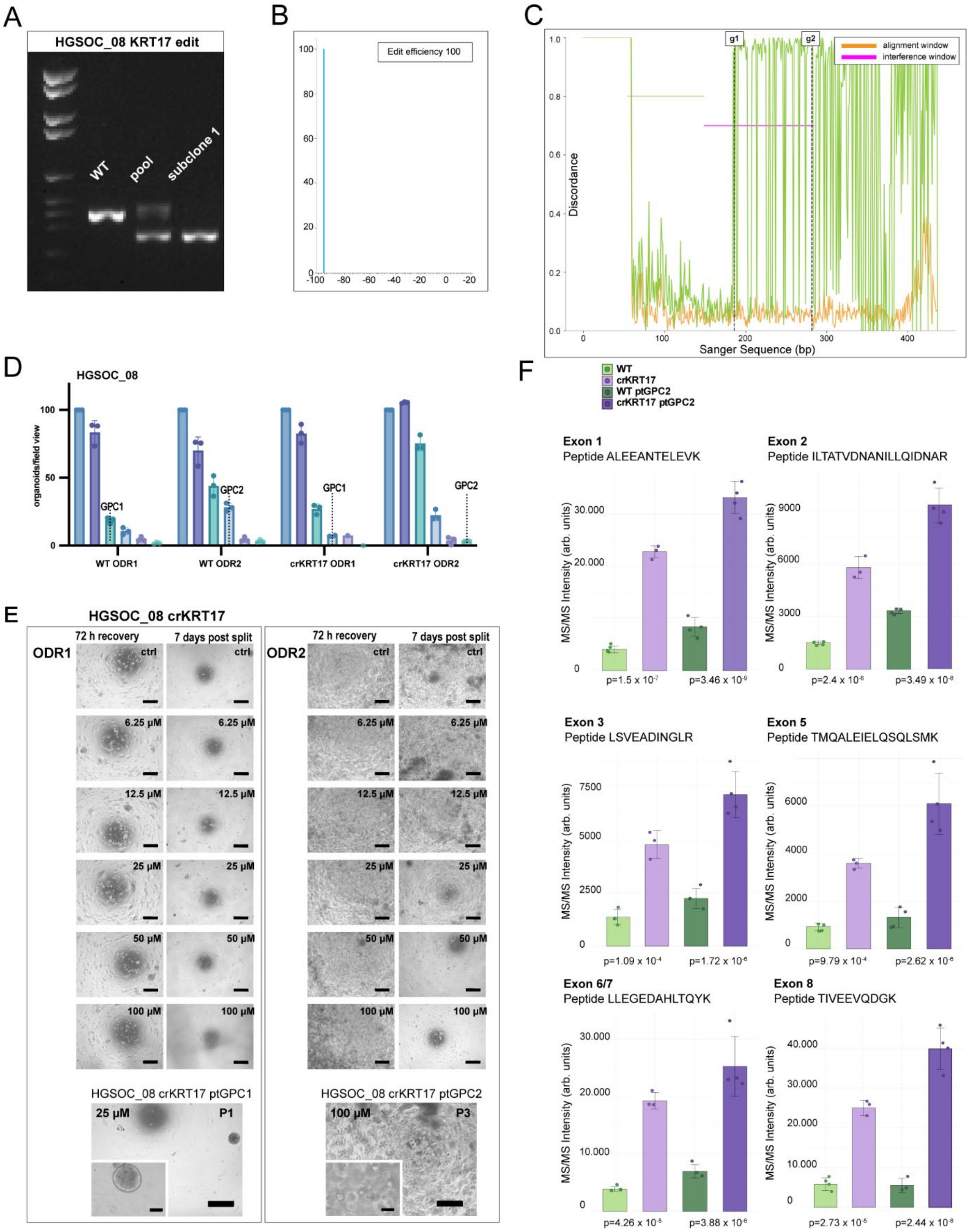

Figure S4

Truncated KRT17 protein, following CRISPR/Cas9 edit, is induced by carboplatin, leading to resistance, related to Figure 4. (A) PCR gel of KRT17 amplicon showing cleavage in the CRISPR/Cas9 edited sample. (B) and (C) discordance plot and sequence comparison (trace)

between WT and edited line generated by the ICE CRISPR analysis tool (Editco). (D) PDO count of WT and crKRT17 line at ODR1 and ODR2 with GPCs (dotted line). (E) Comparative phase contrast images of crKRT17 PDO lines during ODR1 and ODR2 testing and 72 h post-recovery from carboplatin, 7 days post-split (P0), as well as the established long-term stable line at respective GPCs. Scale bars, 500 and 100 µm (zoom in, lower left). (F) Mass spec data showing significant upregulation of peptides representing all other exons of the KRT17 gene in crKRT17 edited organoids, which is further strongly induced by carboplatin challenges (ODR testing). Error bars, mean ±SEM.

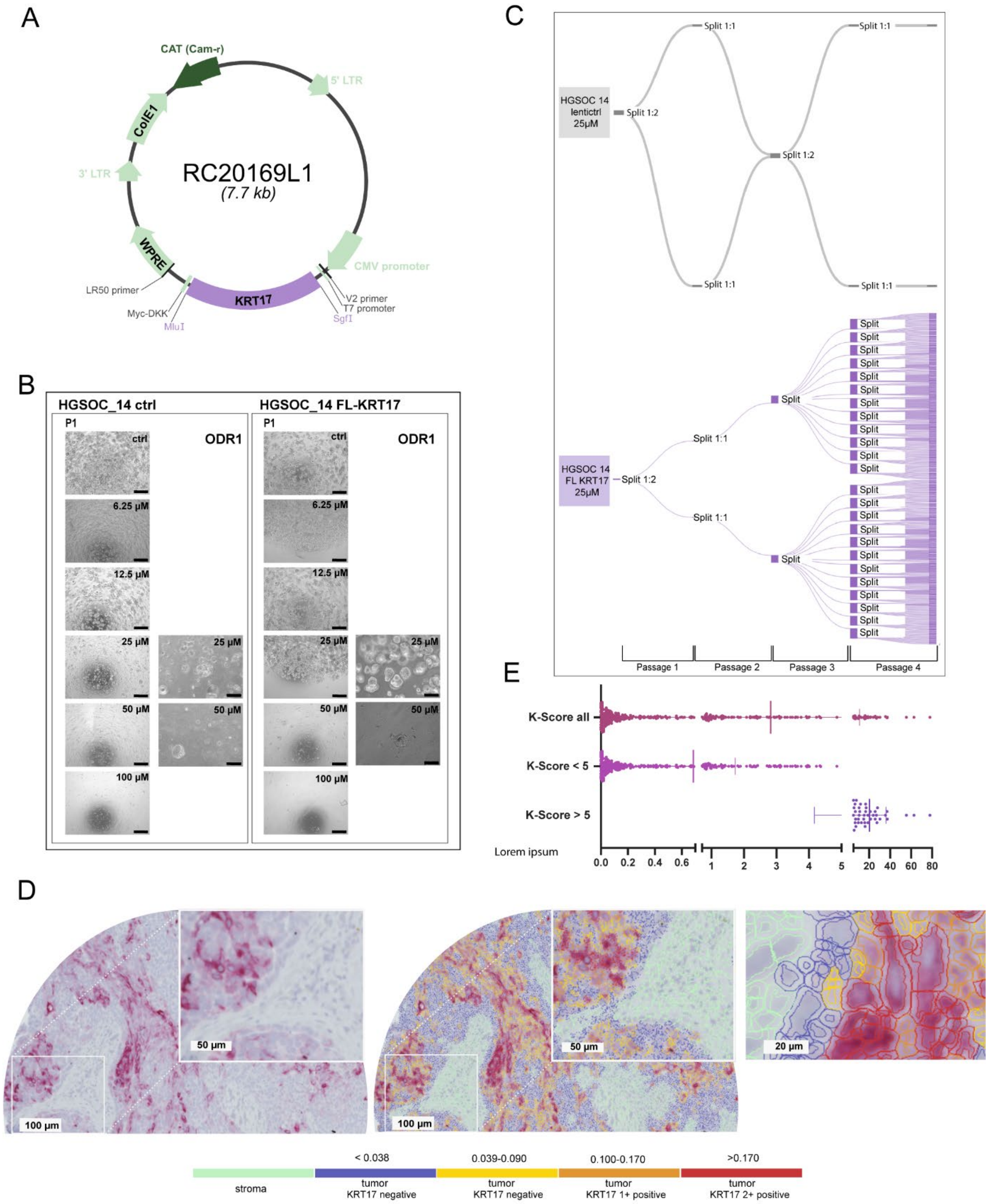

Figure S5

Overexpression of KRT17 drives platinum resistance and is a negative prognostic factor in HGSOC patients, related to Figures 4 and 5.

(A) Plasmid map showing the organization of the transduction vector containing the full-length KRT17 open-reading frame. (B) Phase contrast images showing differences in the organoid count and morphology of transduced lines in P1 of the ODR1 test. (C), Sankey diagram illustrating the growth advantage acquired by FL KRT17 organoids in comparison to the control vector line, after exposure to carboplatin and ODR testing. (D) Exemplary bright field images showing object recognition and signal scoring by the trained Qpath algorithm. (E) Dot plot illustrating the distribution of K-scores of all patient samples of the HGSOC TMA.

Data S1/Methods S1

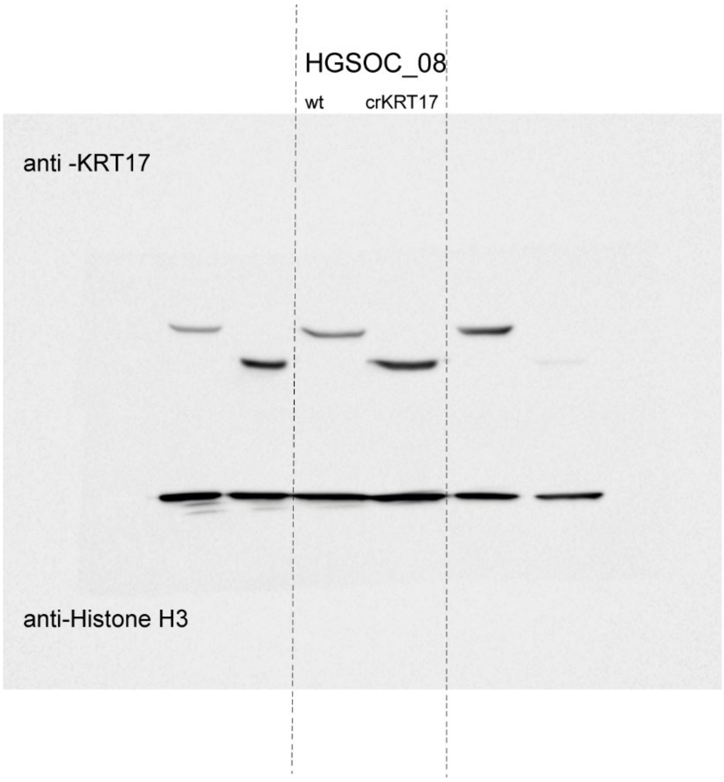

Related to Figure 4 B

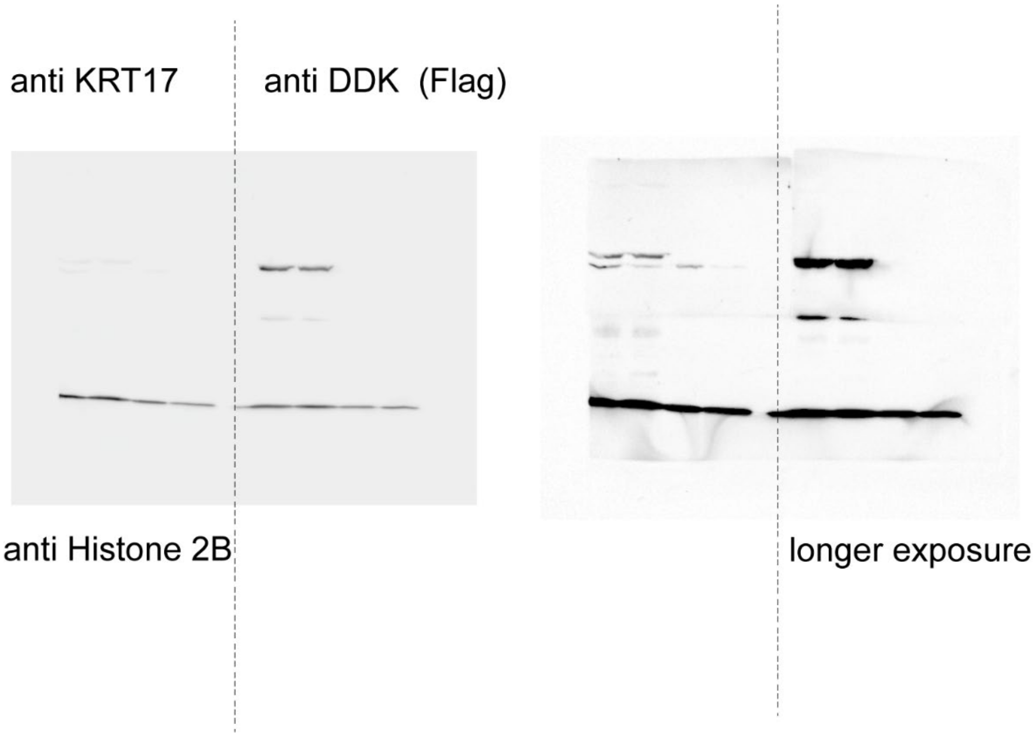

Related to Figure 4 J

Data S1/Methods S1: Unprocessed Western blot membrane images obtained after ECL detection.
